# The involvement of the trisulfur radical anion in electron-catalyzed sulfur insertion reactions: facile synthesis of benzothiazine derivatives under transition metal-free conditions†

**DOI:** 10.1039/c6sc00240d

**Published:** 2016-03-11

**Authors:** Zheng-Yang Gu, Jia-Jia Cao, Shun-Yi Wang, Shun-Jun Ji

**Affiliations:** a Key Laboratory of Organic Synthesis of Jiangsu Province, College of Chemistry, Chemical Engineering and Materials Science & Collaborative Innovation Center of Suzhou Nano Science and Technology, Soochow University Suzhou 215123 China shunjun@suda.edu.cn shunyi@suda.edu.cn

## Abstract

An efficient and practical synthesis of benzothiazine by K_2_S initiated sulfur insertion reaction with enaminones *via* electron catalysis is developed. This protocol provides a new, environment-friendly and simple strategy to construct benzothiazine derivatives *via* formation of two C–S bonds under transition metal-free, additive-free and oxidant-free conditions. K_2_S not only provides the sulfur insertion source, but also ignites the reaction through the formation of a trisulfur radical anion and electrons in DMF.

## Abstract 2

An efficient and practical synthesis of benzothiazine by K_2_S initiated sulfur insertion reaction with enaminones *via* electron catalysis is developed.

## Graphical abstract

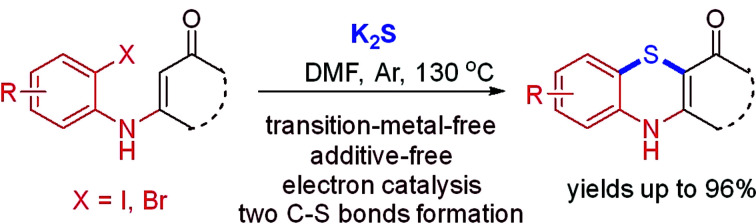

Organosulfur heterocycles have been widely used as functional materials, pharmaceuticals, and synthetic intermediates. 1,4-Benzothiazine derivatives are present in natural products, biologically relevant compounds, and other functional molecules (Fig. 1).^1^ During the past decade, a number of new synthetic protocols for 1,4-benzothiazine construction based on transition metal-catalyzed reactions have been well developed.^2^ The development of new methods to construct 1,4-benzothiazine derivatives *via* C–S bonds formation under transition metal-free conditions is highly desirable.

**Fig. 1:**
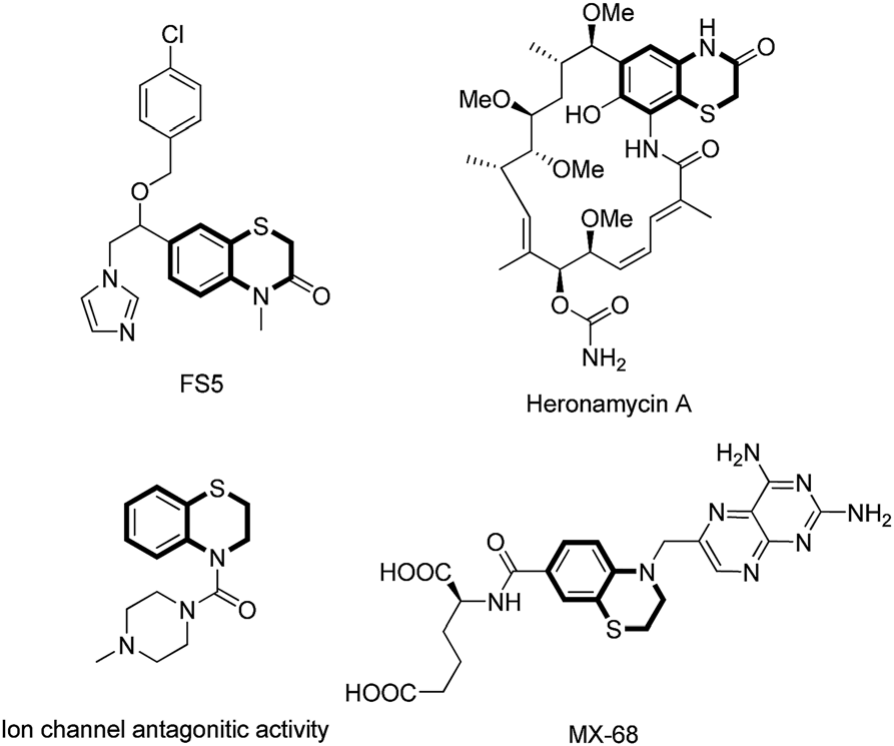
Representative bioactive 1,4-benzothiazine derivatives.

Sulfur-centered radicals such as thiyl radicals and sulfonyl radicals (RSO_2_˙) have gained much interest for their special activities and applications in organic synthesis.^3^ However, other sulfur-centered radicals such as pentafluorosulfanyl radicals (F_5_S˙),^4*a*^ thiocyanato (NCS˙),^4*b*^ and trisulfur radical anion (S_3_˙^−^)^4*c*^ gained less attention for their few applications in organic reactions.^4^ S_3_˙^−^ can be easily formed by the reaction of elemental sulfur with KOH in DMF at room temperature.^5^ Although S_3_˙^−^ species has been known for more than 40 years,^5^ applications for the synthesis of organosulfur compounds have been very limited.^6^ The development of new reactions involving S_3_˙^−^ and its further applications are still great challenges.

Sulfur reagents such as K_2_S have been widely used in transition-metal catalyzed sulfur insertion reactions.^7^ However, there are no reports utilizing K_2_S to initiate aryl radicals or radical anions from aryl halides under transition-metal free conditions (Scheme 1). Herein, we report a K_2_S initiated sulfur insertion reaction with enaminones to construct benzothiazine derivatives *via* formation of two C–S bonds and C–X bond cleavage under transition metal-free conditions. As such, the present reaction enables the synthesis of the benzothiazine derivatives under mild conditions and also attracts more attention for trisulfur radical anion (S_3_˙^−^) involved reactions under transition metal-free conditions.

**Scheme 1:**
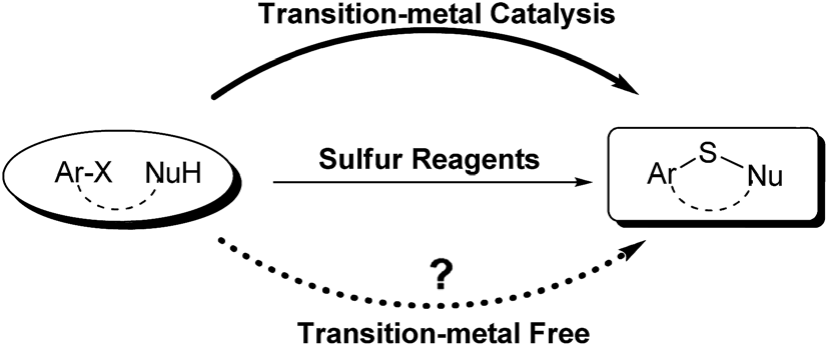
Approaches for aryl sulfide.

Initially, the model reaction of 3-((2-iodophenyl)amino)-5,5-dimethylcyclohex-2-enone 1a and K_2_S 2a was performed in DMF at 110 °C for 12 h catalyzed by 10 mol% CuI in the presence of 20 mol% of I_2_ under Ar atmosphere. The [5 + 1] cyclization product 4*H*-benzo[*b*][1,4]thiazine derivative 3a was obtained in 65% liquid chromatography yield (LC-yield). The structure of 3a was confirmed by NMR, IR, HRMS and X-ray analysis (Fig. 2).

**Fig. 2:**
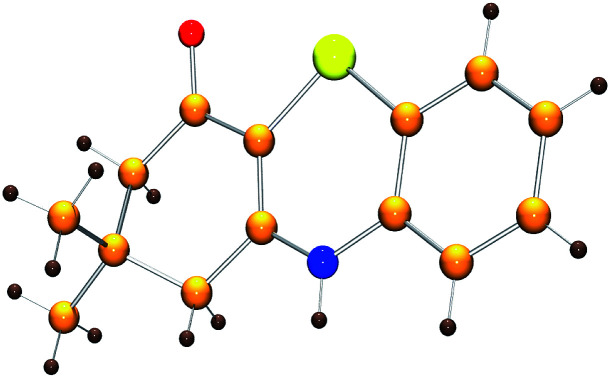
Crystal structure of 3a.

We further screened the reaction conditions and found that the reaction also proceeded even without the copper salt and iodine (for details see ESI†). Then different solvents such as CH_3_CN, 1,4-dioxene, 1,2-dichloroethane (DCE), toluene, tetrahydrofuran (THF), dimethyl sulfoxide (DMSO), *N*,*N*-dimethylformamide (DMF) and H_2_O were tested for the reaction of 1a and 2a at 130 °C for 12 h under Ar atmosphere. As shown in Fig. 3, CH_3_CN, 1,4-dioxene could give moderate yields of 3a. Trace product 3a could be detected when the reaction was carried out in DCE, toluene, THF and H_2_O, respectively. Gratifyingly, the LC-yield of 3a was dramatically increased to 75% by using DMSO as solvent. It should be noted that the reaction proceeded smoothly to give 3a in 90% LC-yield when DMF was used.

**Fig. 3:**
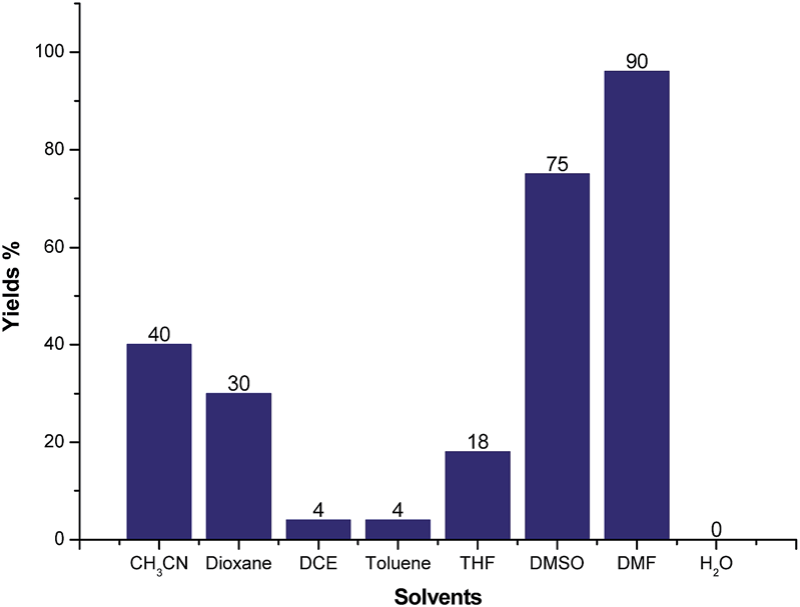
The effect of different solvents. *Reaction conditions*: 1a (0.5 mmol), 2a (0.6 mmol), solvent (3 mL) at 130 °C, 12 h under Ar atmosphere. The yields were determined by LC analysis using biphenyl as the internal standard.

Then other sulfur reagents such as cyclo-S_8_, Na_2_S·9H_2_O, NaHS·H_2_O, Na_2_S_2_O_3_ were applied in this reaction under transition metal-free conditions (Table 1). The reaction of 1a with Na_2_S·9H_2_O occurred smoothly to give 3a in 80% LC-yield. NaHS·H_2_O also showed a moderate activation with 1a in this reaction to give 3a 60% LC-yield. 3a could also be obtained in 27% LC-yield when the sulfur was used. Only trace amount of 3a was detected when 1a reacted with Na_2_S_2_O_3_ under the identical conditions.

**Table 1:** Yields of 3a from 1a for various sulfur reagents^a^

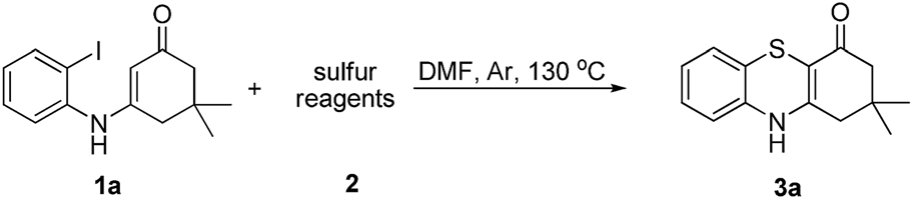	
Reagent	Yield (%)
K_2_S	80
Cyclo-S_8_	27
Na_2_S·9H_2_O	80
NaHS·H_2_O	60
Na_2_S_2_O_3_	Trace

a *Reaction conditions*: 1a (0.5 mmol), 2 (0.6 mmol), DMF (3 mL) at 130 °C, 12 h under Ar atmosphere.

To explore the potential applications of this method, a variety of enaminones 1 were examined (Table 2). Electron-donating substituents on the phenyl ring of enaminone such as –Me, –OMe, promoted the cross-coupling product 3b and 3c in excellent yield (95, 88%). The substitution pattern of the chlorine group made some difference to the reaction outcome (3d, 3e and 3f). The reaction of 3-(4-fluorophenylamino)-5,5-dimethylcyclohex-2-enone 1g with 2a also led to the desired product 3g in 85% yield. Electron-withdrawing groups, such as NO_2_, CF_3_, also worked well and the desired products (3h, 3i) were isolated in excellent yields, too. The reactions of other substituted enaminones 1i–l with 2a furnished the products 3i–l in moderate to excellent yields (47–96%). Unfortunately, when the unstable enaminone (*E*)-4-(phenylamino)pent-3-en-2-one 1n was applied to the reaction, trace product 3n could be detected.

**Table 2:** Synthesis of 4*H*-benzo[*b*][1,4]thiazine derivatives^a^

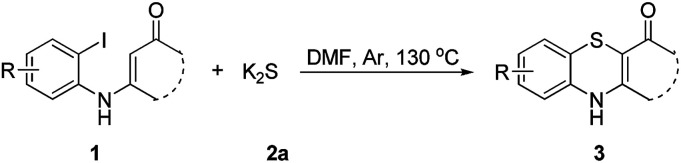
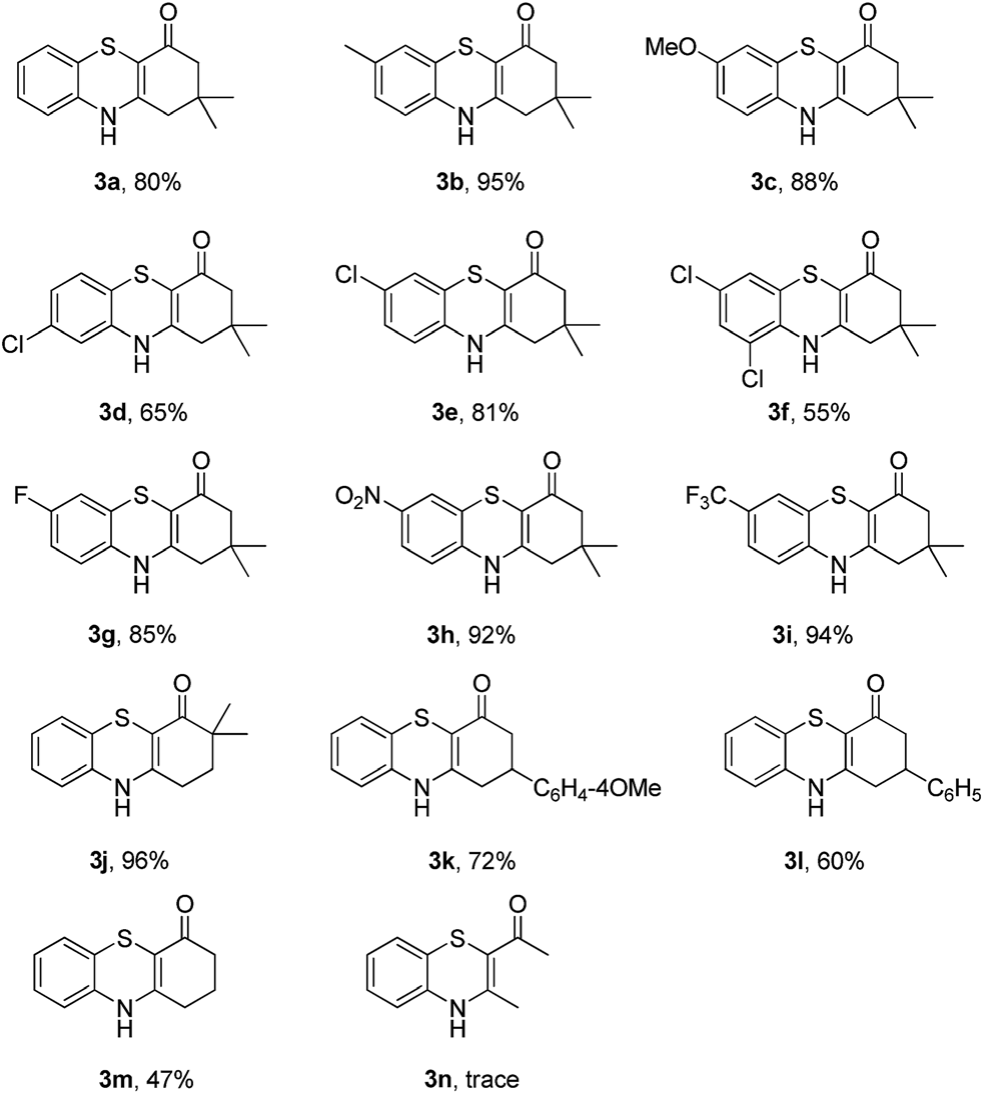

a *Reaction conditions*: 1 (0.5 mmol), 2a (0.6 mmol), DMF (3 mL) at 130 °C, 12 h under Ar atmosphere.

Subsequently, enaminones 3-((2-chlorophenyl)amino)-6,6-dimethylcyclohex-2-enone 1a-Cl and 3-((2-bromophenyl)amino)-6,6-dimethylcyclohex-2-enone 1a-Br instead of 1a-I were applied to reaction with 2a. The results indicated that chloro-functionalized enaminone 1a-Cl showed poor reactivity but bromo-functionalized enaminone 1a-Br showed competitive reactivity compared to iodo-functionalized enaminone 1a-I and resulted in 3a in 94% yield (Scheme 2). Some other bromo-functionalized enaminones reacted with 2a also has been investigated (Table 3). The reactions of 3-((2,5-dibromophenyl)amino)-5,5-dimethylcyclohex-2-enone and 3-((2,6-dibromophenyl)amino)-5,5-dimethylcyclohex-2-enone with K_2_S were explored, and the desired products 3o and 3p were observed in 80% and 68% yields, respectively, leaving one bromide and another bromide substituents untouched. However, the reaction of 5,5-dimethyl-3-((2,4,6-tribromophenyl)amino)cyclohex-2-enone failed to isolate the corresponding product 3q. The 3-(2-iodo-4-nitrophenylamino)-5,5-dimethylcyclohex-2-enone 1h-Br promoted the cross-coupling product 3h in 48% yield. Other substituted enaminones (1j-Br to 1m-Br) reacted with 2a also furnished the desired products 3j-m in moderate yields (50% to 88%).

**Scheme 2:**
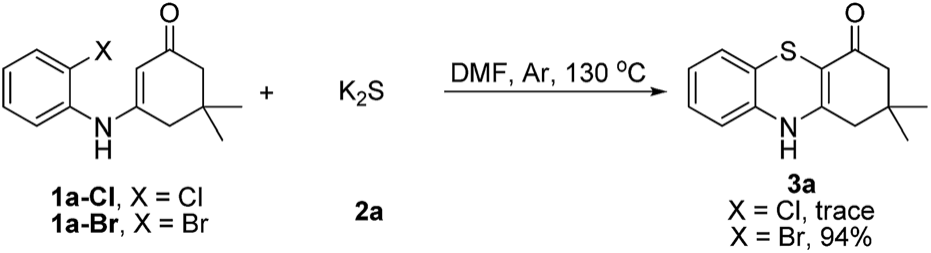
The reaction with 2a.

**Table 3:** Synthesis of 4*H*-benzo[*b*][1,4]thiazine derivatives^a^

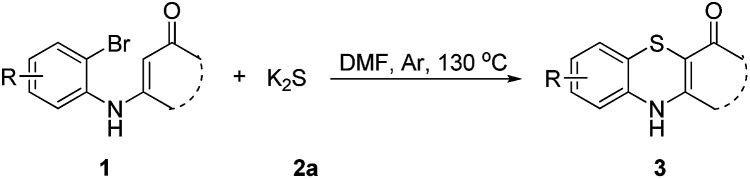
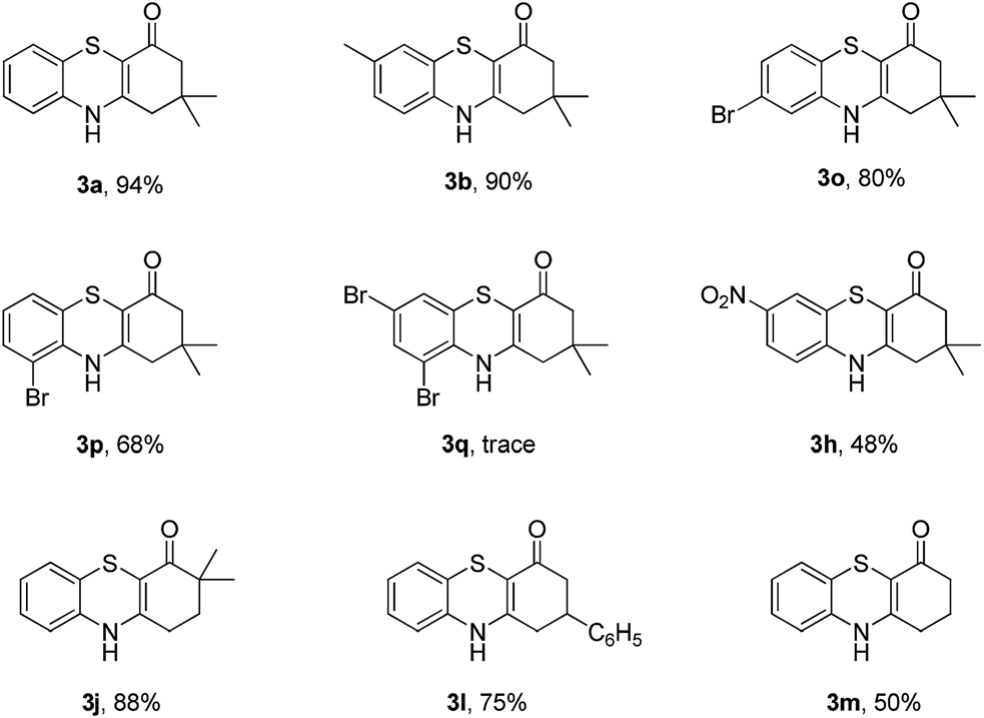

a *Reaction conditions*: 1 (0.5 mmol), 2a (0.6 mmol), DMF (3 mL) at 130 °C, 12 h under Ar atmosphere.

Then, an attempt was made to achieve the efficient synthesis of 2-phenyl-4*H*-thiochromen-4-one derivatives with 2′-bromochalcones 4 as the starting materials (Table 4). The reaction of 2′-bromochalcone 4a progressed well and gave the corresponding product 5a in 51% yield. The 2′-bromochalcones with methyl, chloro and fluoro substituents, which could be useful for further derivatization, were found to be suitable for this reaction (5b–d). Besides aryl groups, substrates with thienyl group 4e was also found to be appropriate for this reaction, and the corresponding product 5e was obtained in 60% yield.

**Table 4:** Synthesis of 2-aryl-4*H*-thiochromen-4-one derivatives^a^

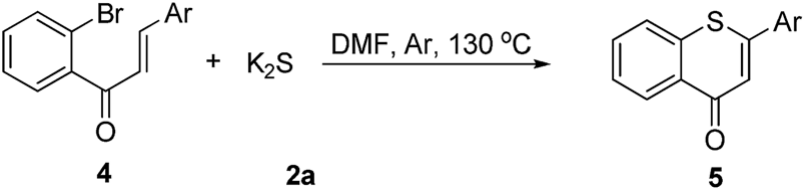
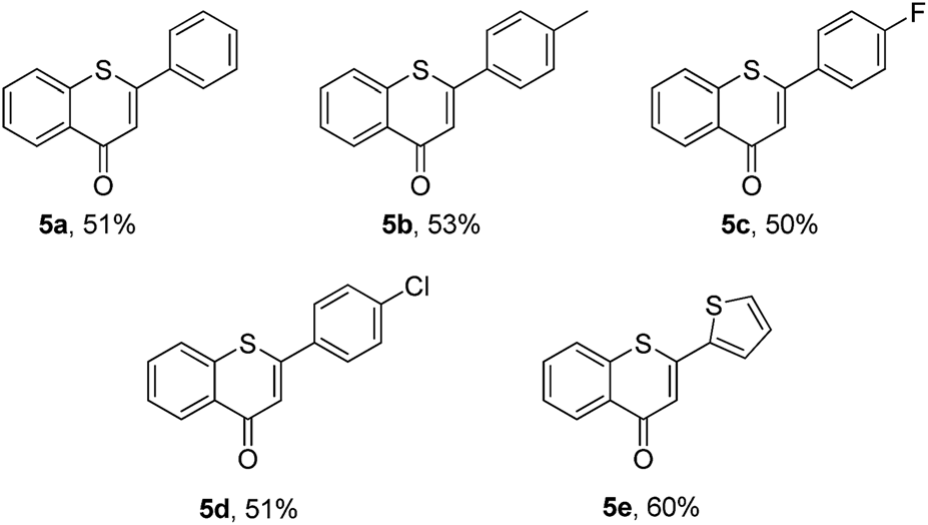

a *Reaction conditions*: 4 (0.5 mmol), 2a (0.6 mmol), DMF (3 mL) at 130 °C, 12 h under Ar atmosphere.

To explore the plausible mechanism of this reaction, we first analyzed K_2_S using inductively coupled plasma atomic absorption spectroscopy (ICP-AAS). Indeed, 2–10 ppm of Cu, Pd, Fe species were detected in K_2_S although its purity is >99.99%. It was found that adding 1000 times amounts of these metal salts to the reaction system did not obviously enhance the reaction rate, which indicated that this reaction was not transition-metal catalyzed and the radical addition pathway was an apparent consideration to the mechanism. Therefore, radical-trapping experiments were performed as shown in Scheme 3. When 1.0 equiv. of TEMPO was added to the reaction the yield of 3a was reduced to 47%. As the amount of TEMPO was increased to 4.0 equiv., the reaction was almost fully suppressed. This observation indicated that a radical pathway might be involved in the reaction.

**Scheme 3:**
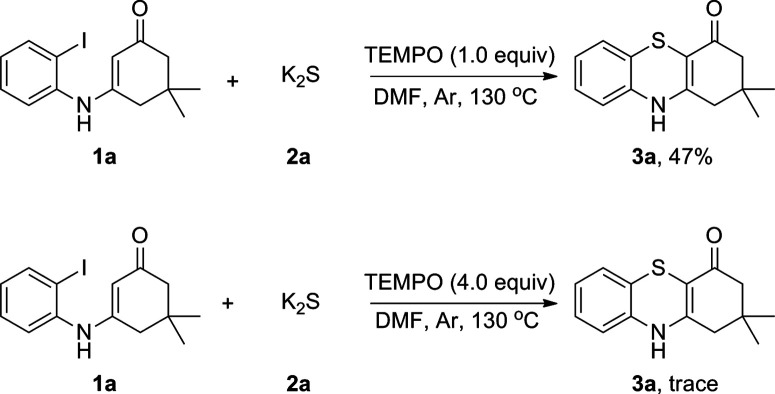
Radical-trapping experiments.

To gain some insight into the interaction between K_2_S and DMF or other solvents, an electron paramagnetic resonance (EPR) experiment was carried out (Fig. 4). A strong single EPR signal was observed in DMF solution of K_2_S at room temperature (deep blue line). When the solvent was changed to DMSO, the EPR signal was significantly attenuated (purple line). As for the other solvents, almost no EPR signal was observed. Lelieur found similar EPR signals in liquid ammonia solution of sulfur, and identified it as the trisulfur radical anion (S_3_˙^−^).^8^ Gratifyingly, our EPR parameter (*g* = 2.02) is very similar with the reported one detected by the DMF solution of Na_2_S·9H_2_O and elemental sulfur at room temperature.^6*b*^ So we suspected that a free radical S_3_˙^−^ might be generated from the solution of K_2_S in DMF.

**Fig. 4:**
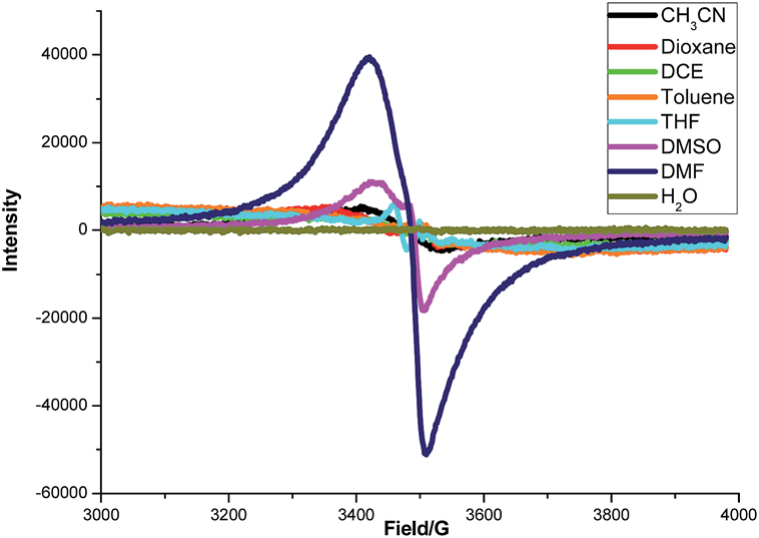
EPR spectra of solutions of K_2_S (0.6 mmol) in various solvents (3 mL) at 298 K.

We further confirmed the presence of trisulfur radical anion (S_3_˙^−^) by the UV-visible spectra and Raman spectra studies (Fig. 5). From the UV-visible spectra, we could detect a characteristic absorption peak at about 550–700 nm wavelength in the DMF solutions of K_2_S or Na_2_S·9H_2_O and elemental sulfur. In addition, the Raman spectra results also gave us some strong evidence of the trisulfur radical anion (S_3_˙^−^).^9^ The 531 cm^−1^ peak (*ν*_1_) corresponds to the symmetric S–S stretching. The resonance phenomenon is induced *via* the absorption of S_3_˙^−^ by the laser radiation, which results in the high-order overtones (2*ν*_1_ ≈ 1068 cm^−1^, 3*ν*_1_ ≈ 1597 cm^−1^, 4*ν*_1_ ≈ 2124 cm^−1^) for the enhancement effects.^10^

**Fig. 5:**
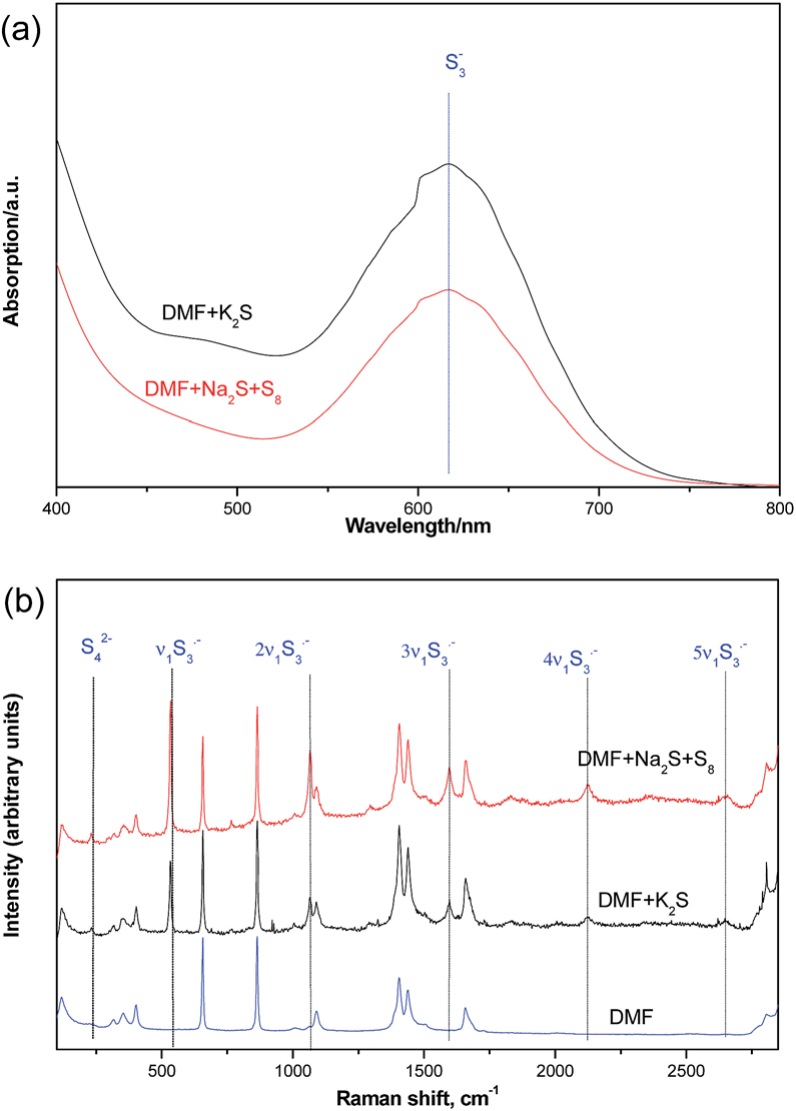
UV-visible spectra (a) and Raman spectra (b) (632.8 nm) studies of interaction between K_2_S and DMF.

We also found that the reaction takes place to give similar results under an argon atmosphere or in air. This observation indicated that DMF might be the oxidant for this reaction instead of oxygen or other oxidants. In order to prove this proposal, we tried the reaction of 1a with 2a in d^7^-DMF (Fig. 6). From the crude ^1^H NMR spectrum of this reaction, it was found that d^7^-DMF was reduced to give G′ (for further details see ESI†). This result indicates that S_3_˙^−^ was formed *via* the oxidation of S^2−^ by DMF, which is different with the one in Lei's work (S_3_˙^−^ was obtained by the disproportionation reaction of sulfur with Na_2_S·9H_2_O).^6*b*^

**Fig. 6:**
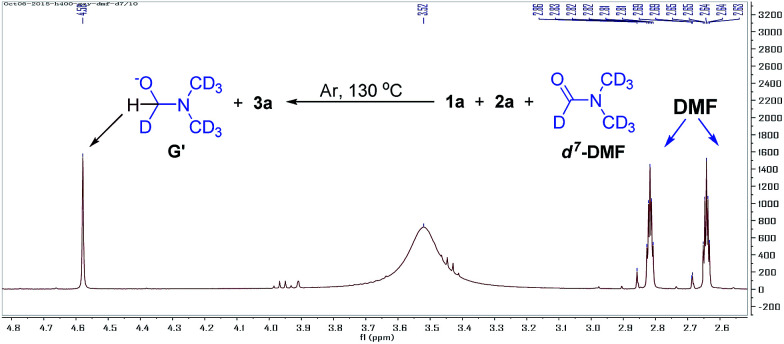
^1^H NMR spectrum of the reaction of 1a with 2a in *d*^7^-DMF.

Based on the reported literatures and above results, a S_RN_1 type electron catalysis mechanism^11^ is proposed in Scheme 4. DMF activates the electron-donating agent K_2_S to give S_3_˙^−^ radical anion, radical anion A and electrons. An aryl halide radical anion B is generated from enaminone 1a by single electron transfer (SET).^12^ The elimination of I^−^ leads to an aryl radical C (the protonated species could be detected by LC-MS^13^), the radical C reacts with S_3_˙^−^ to form the intermediate D. The homolysis of intermediate D gives S_2_˙^−^ and a thiyl radical E, which subsequently undergoes intramolecular radical addition to afford the radical F. We propose that there are two possible pathways for the oxidation of S_2_˙^−^ to give S_3_˙^−^. One possibility is that S_2_˙^−^ dimerizes to afford the dianion S_4_^2−^, which then disproportionates to give blue S_3_˙^−^.^14^ The other possibility is that S_2_˙^−^ reacts with S^2−^ to afford S_3_˙^−^ and the electrons. Following the electron transfer of F with A, anion G and 3a are formed.

**Scheme 4:**
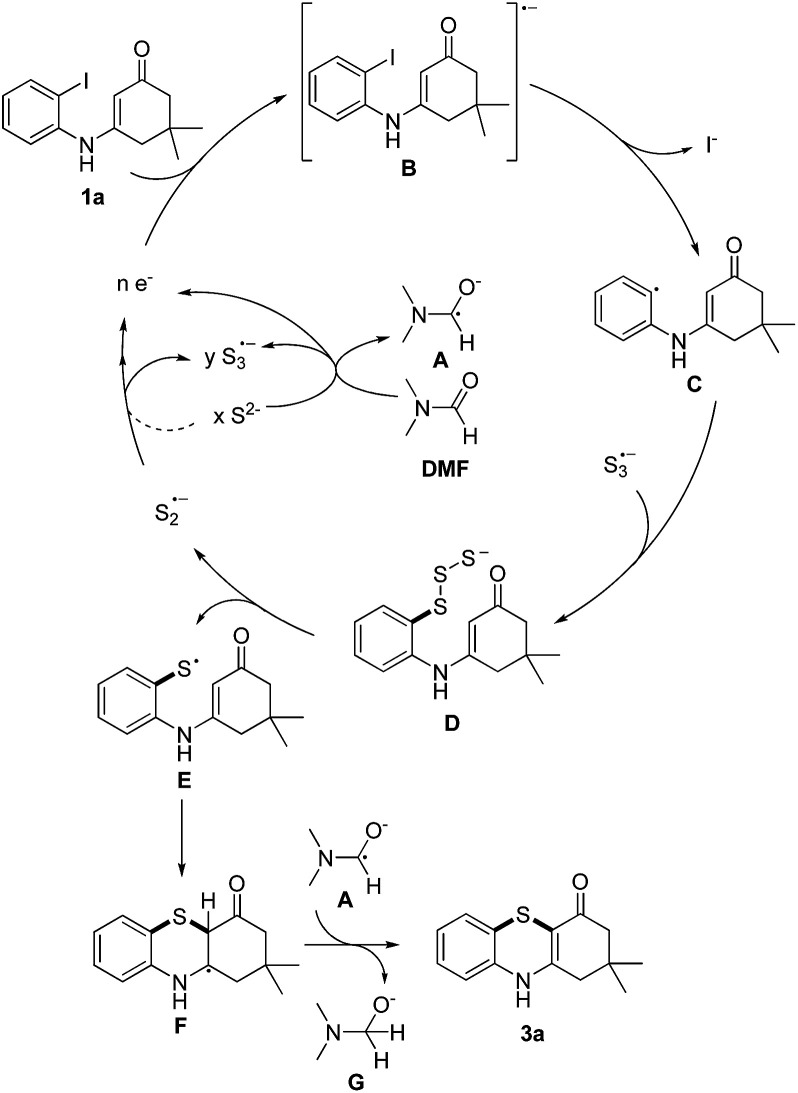
Plausible mechanism.

In summary, we have developed a K_2_S initiated and electron-catalyzed sulfur insertion reaction with enaminones to construct the [5 + 1] cyclization product 4*H*-benzo[*b*][1,4]thiazine and 2-aryl-4*H*-thiochromen-4-one derivatives. This protocol provides a new, environment-friendly and simple strategy to the synthesis of the 4*H*-benzo[*b*][1,4]thiazine and 2-aryl-4*H*-thiochromen-4-one derivatives under transition metal-free, additive-free and oxidant-free conditions. The presence of the trisulfur radical anion was proven by EPR spectroscopy, and reasonable mechanisms have been proposed. Further investigations of the trisulfur radical anion triggered electron-catalyzed reactions under transition metal-free conditions are currently under study in our laboratory.

## Supplementary Material

SC-007-C6SC00240D-s001

SC-007-C6SC00240D-s002
