# Supplementary material for: The involvement of the trisulfur radical anion in electron-catalyzed sulfur insertion reactions: facile synthesis of benzothiazine derivatives under transition metal-free conditions
Source: Chem Sci. 2016 Mar 11;7(7):4067–72. doi: 10.1039/c6sc00240d (PMC6013918; doi:10.1039/c6sc00240d)
Supplement: SC-007-C6SC00240D-s001 [file SC-007-C6SC00240D-s001.pdf]

Electronic Supplementary Material (ESI) for Chemical Science.
This journal is © The Royal Society of Chemistry 2016

Supporting Information for

The Involvement of the Trisulfur Radical Anion in Electron-Catalyzed Sulfur Insertion Reactions: Facile Synthesis of Benzothiazine Derivatives under Transition Metal-free Conditions

Zheng-Yang Gu, Jia-Jia Cao, Shun-Yi Wang,* and Shun-Jun Ji*

Key Laboratory of Organic Synthesis of Jiangsu Province, College of Chemistry, Chemical Engineering and Materials Science & Collaborative Innovation Center of Suzhou Nano Science and Technology, Soochow University, Suzhou 215123, China

E-mail: shunyi@suda.edu.cn; shunjun@suda.edu.cn

Table of Contents

Experimental Section and crystal structures---------------------------------------S2-S6

Characterization Data of Compounds **3a-5e**---------------------------------------S7-S13

Copies of ^{1}H and ^{13}C NMR Spectra for Compounds **3a-5e**----------------------S14-S33

X-ray crystallographic data of **3a** --S34-S42

Experimental Section

General

Melting points were recorded on an Electrothermal digital melting point apparatus and were uncorrected. IR spectra were recorded on a Bruker Tensor 27 spectrophotometer. ^{1}H NMR and ^{13}C NMR spectra were recorded on a Bruker 400 MHz (^{1}H NMR) and 400 MHz (^{13}C NMR) spectrumeter using $CDCl_3$ or DMSO-d_6 as solvent and TMS as internal standard. EPR spectra were recorded on a Bruker EXM-10/2 spectrophotometer. UV spectra were recorded on a UV1102 spectrophotometer. Raman spectra were recorded on a Horiba Jobin Yvon LabRam HR800 spectrophotometer. High resolution mass spectra were obtained using GCT-TOF instrument with ESI source or EI source.

Typical procedure for benzothiazine of K_2S with enaminones

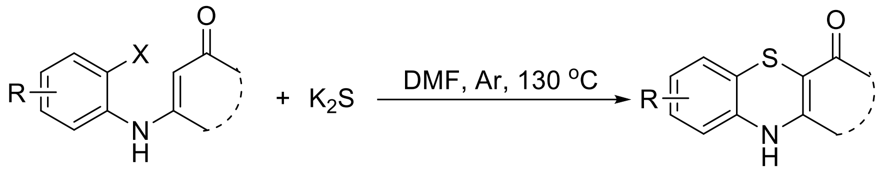

1 **2** **3**

A mixture of enaminones **1** (0.5 mmol) and K_2S **2** (0.6 mmol) and DMF (3 mL) were added into a flask and stirred at 110 °C under Ar atmosphere. Then the mixture was vigorously stirred under reflux conditions monitored by TLC analysis (about 12 h). After removing the solvents in vacuo, the residue was directly purified by flash column chromatography by using ethyl acetate and petroleum ether as eluents to afford pure product **3**.

Typical procedure for 2- aryl -4*H*-thiochromen-4-one Derivatives of K_2S with 2'-bromochalcones

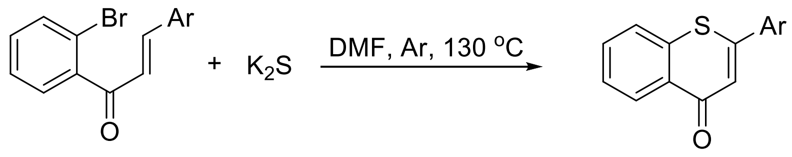

4 **2a** **5**

A mixture of 2'-bromochalcones **4** (0.5 mmol) and K_2S **2** (0.6 mmol) and DMF (3 mL) were added into a flask and stirred at 110 °C under Ar atmosphere. Then the mixture was vigorously stirred under reflux conditions monitored by TLC analysis (about 12 h). After removing the solvents in vacuo, the residue was directly purified by flash column chromatography by using ethyl acetate and petroleum ether as eluents to afford pure product **5**.

EPR Studies of Interaction between K_2S and DMF

A dried tube equipped with a stir bar was loaded with K_2S (0.50 mmol) in 3.0 mL DMF was stirred at 25 ℃. After 30 mins, the solution sample was taken out into a small tube

and analyzed by EPR. EPR spectra was recorded at room temperature on EPR spectrometer operated at 9.852 GHz. Typical spectrometer parameters are shown as follows, scan range: 1000 G; center field set: 3400 G; scan time: 35 s.

EPR Studies of Interaction between K_2S and H_2O

A dried tube equipped with a stir bar was loaded with K_2S (0.50 mmol) in 3.0 mL H_2O was stirred at 25 ℃. After 30 mins, the solution sample was taken out into a small tube and analyzed by EPR. EPR spectra was recorded at room temperature on EPR spectrometer operated at 9.852 GHz. Typical spectrometer parameters are shown as follows, scan range: 1000 G; center field set: 3400 G; scan time: 35 s.

UV spectra Studies of Interaction between K_2S and DMF

A dried tube equipped with a stir bar was loaded with K_2S (0.50 mmol) in 3.0 mL DMF was stirred at 25 ℃. Then, the solution sample was taken out into a small tube and analyzed by UV spectrometer operated. Typical spectrometer parameters are shown as follows, scan range: 400 nm; wavelength from 400 nm-800 nm; Scan speed: 800 nm/min

UV spectra Studies of Interaction between Na_2S, S and DMF

A dried tube equipped with a stir bar was loaded with Na_2S (0.50 mmol), S (0.50 mmol) in 3.0 mL DMF was stirred at 25 ℃. Then, the solution sample was taken out into a small tube and analyzed by UV spectrometer operated. Typical spectrometer parameters are shown as follows, scan range: 400 nm; wavelength from 400 nm-800 nm; Scan speed: 800 nm/min

Raman spectra Studies of Interaction between K_2S and DMF

A dried tube equipped with a stir bar was loaded with K_2S (0.50 mmol) in 3.0 mL DMF was stirred at 25 ℃. Then, the solution sample was taken out into a small tube and analyzed by Raman spectrometer operated. Typical spectrometer parameters are shown as follows, scan time: 50 s; wavelength 632.8 nm.

Raman spectra Studies of Interaction between Na_2S, S and DMF

A dried tube equipped with a stir bar was loaded with Na_2S (0.50 mmol), S (0.50 mmol) in 3.0 mL DMF was stirred at 25 ℃. Then, the solution sample was taken out into a small tube and analyzed by Raman spectrometer operated. Typical spectrometer parameters are shown as follows, scan time: 50 s; wavelength 632.8 nm.

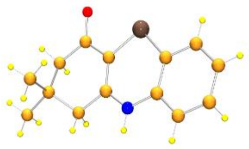

Figure 1 Crystal Structure of 3a

Screening of Reaction Conditions: Effects of Catalyst, Additive and Solvent[a]

Entry	Cat. (10 mol%)	Solvent	Add. (x mol%)	T (℃)	Yield(%)[b]
1	CuI	DMF	I_2 (20)	110	65
2	$Cu(OAc)_2$	DMF	I_2 (20)	110	78
3	$CuCl_2$	DMF	I_2 (20)	110	58
4	Cu_2O	DMF	I_2 (20)	110	60
5	$CuBr_2$	DMF	I_2 (20)	110	38
6	CuBr	DMF	I_2 (20)	110	60
7	$CuSO_4$	DMF	I_2 (20)	110	50
8	$Cu(OAc)_2$	CH_3CN	I_2 (20)	110	39
9	$Cu(OAc)_2$	1,4-dioxene	I_2 (20)	110	39
10	$Cu(OAc)_2$	DCE	I_2 (20)	110	40
11	$Cu(OAc)_2$	THF	I_2 (20)	110	53
12	$Cu(OAc)_2$	Toluene	I_2 (20)	110	8
13	$Cu(OAc)_2$	DMSO	I_2 (20)	110	trace
14	$Cu(OAc)_2$	Xylene	I_2 (20)	110	trace
15	$Cu(OAc)_2$ (10)	DMF	-------	110	67
16	$Cu(OAc)_2$ (10)	DMF	I_2 (10)	110	56
17	$Cu(OAc)_2$ (10)	DMF	I_2 (30)	110	60
18	$Cu(OAc)_2$ (10)	DMF	-------	120	82
19	$Cu(OAc)_2$ (10)	DMF	-------	130	88
20	$Cu(OAc)_2$ (5)	DMF	-------	130	91
21	$Cu(OAc)_2$ (1)	DMF	-------	130	89
22	-----------	DMF	-------	130	87(80)[c]

[a]Reaction Conditions: **1a** (0.5 mmol), **2a** (0.6 mmol), solvent (3 mL) under air atmosphere. [b]The yields were determined by LC analysis using biphenyl as the internal standard. [c]Isolated yields.

The ^{1}H NMR of *d*^{7}-DMF

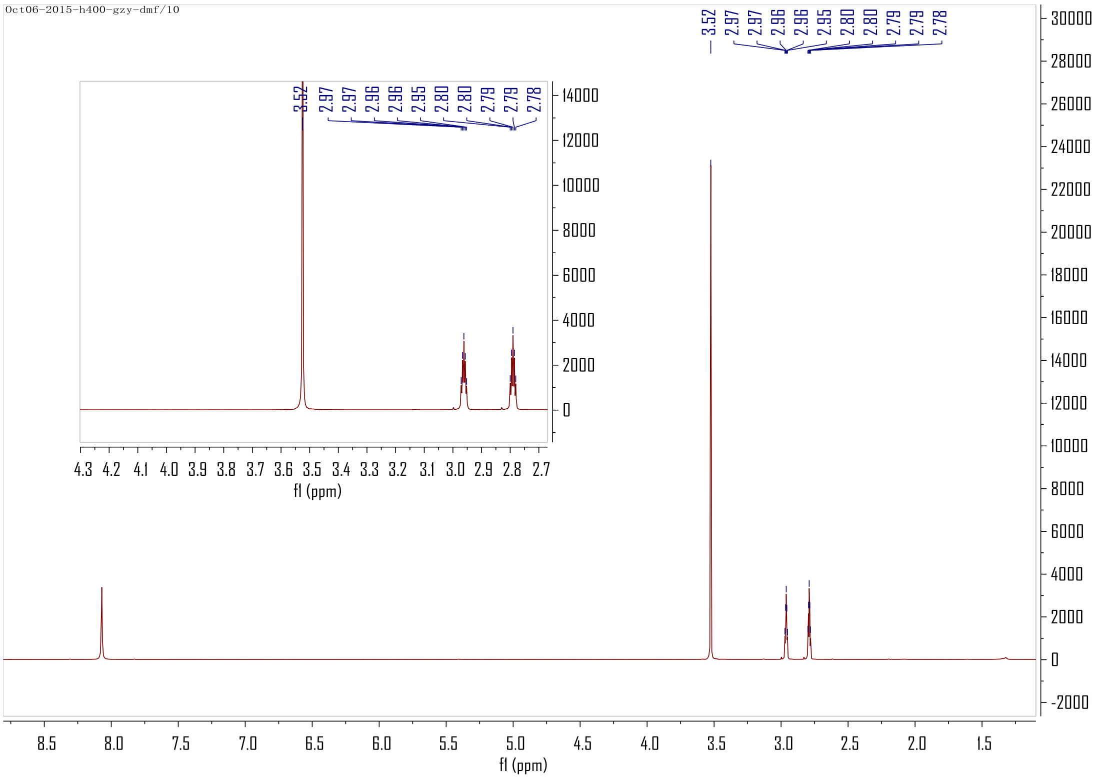

Crude ^{1}H NMR of the reaction of 1a with 2a in *d*^{7}-DMF

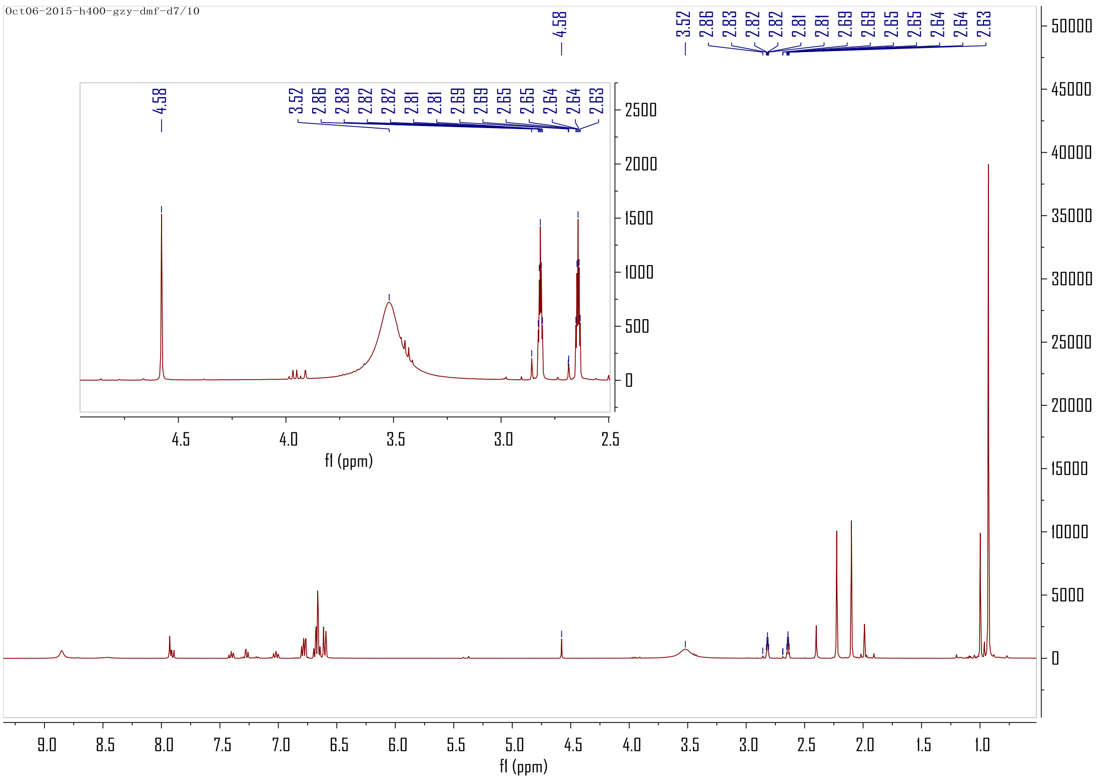

UV spectra studies of interaction between K_2S and DMF

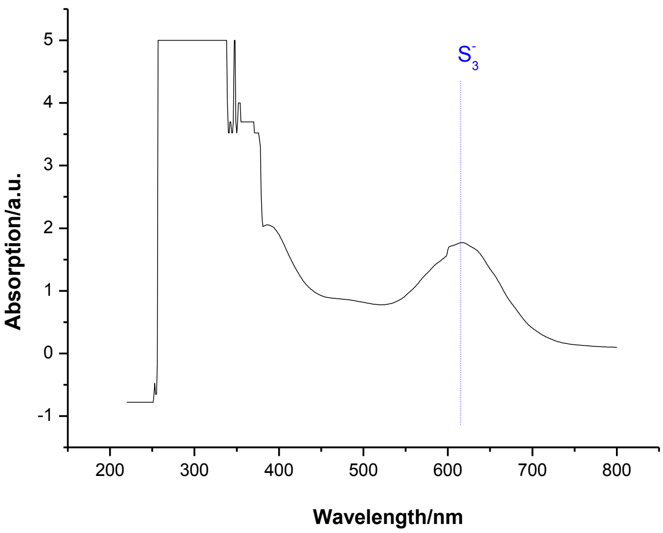

Plausible chemical balance and mechanism

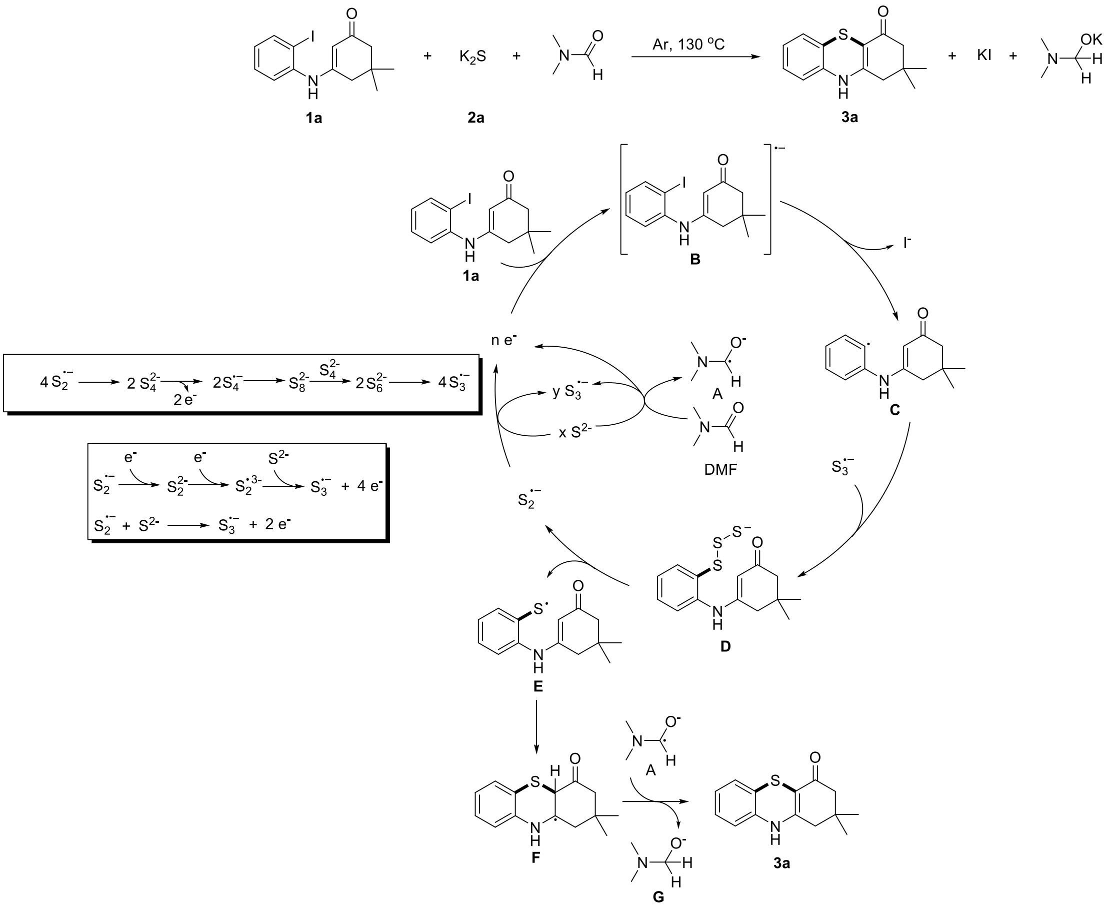

Ar, 130 ºC
1a
2a
3a
B
C
D
E
F
G
A
DMF
n e⁻

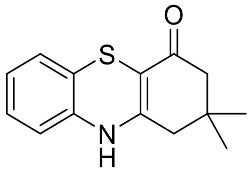

2,2-dimethyl-2,3-dihydro-1H-phenothiazin-4(10H)-one (3a)

Yield=80%. Yellow solid. M.p. 254.4-255.7℃. IR 3247, 2955, 2927, 1611, 1522, 1468, 1306, 746 cm^{-1}. ^{1}H NMR (400 MHz, DMSO) δ 8.85 (s, 1H, N-H), 6.86 (t, *J* = 5.8 Hz, 1H, Ar-H), 6.78 – 6.67 (m, 2H, Ar-H), 6.54 (d, *J* = 7.6 Hz, 1H, Ar-H), 2.20 (s, 2H, -CH_2), 2.15 (s, 2H, -CH_2), 1.00 (s, 6H, -CH_3). ^{13}C NMR (101 MHz, DMSO) δ 189.00, 154.43, 137.16, 127.36, 126.93, 125.02, 120.34, 116.19, 97.03, 50.20, 41.73, 31.97, 28.08 ppm. HRMS (ESI) m/z calculated for $C_{14}H_{15}NOS$, 245.0874; found 245.0871.

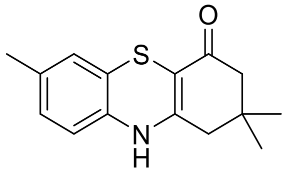

2,2,7-trimethyl-2,3-dihydro-1H-phenothiazin-4(10H)-one (3b)

Yield=95%. Yellow solid. M.p. 283.3-285.6℃. IR 3245, 2956, 2921, 1592, 1560, 1478, 1310, 814, 746 cm^{-1}. ^{1}H NMR (400 MHz, DMSO) δ 8.80 (s, 1H, N-H), 6.66 (d, *J* = 7.8 Hz, 1H, Ar-H), 6.56 (s, 1H, Ar-H), 6.44 (d, *J* = 7.9 Hz, 1H, Ar-H), 2.19 (s, 2H, -CH_2), 2.13 (s, 2H, -CH_2), 2.07 (s, 3H, -CH_3), 0.99 (s, 6H, -CH_3). ^{13}C NMR (101 MHz, DMSO) δ 188.26, 153.74, 133.93, 133.74, 127.08, 126.80, 119.73, 115.61, 96.06, 49.69, 41.25, 31.46, 27.60, 19.98 ppm. HRMS (ESI) m/z calculated for $C_{15}H_{17}NOS$, 259.1031; found 259.1027.

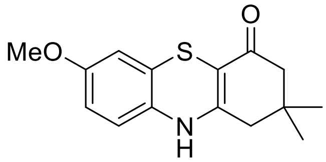

7-methoxy-2,2-dimethyl-2,3-dihydro-1H-phenothiazin-4(10H)-one (3c)

Yield=88%. Yellow solid. M.p. 270.1-273.3℃. IR 3242, 3031, 2965, 1604, 1561, 1478, 1355, 1070, 793 cm^{-1}. ^{1}H NMR (400 MHz, DMSO-*d*$_6$) δ 8.83 (s, 1H, N-H), 6.50 (d, *J* = 8.5 Hz, 1H, Ar-H), 6.44 (dd, *J* = 8.7, 2.7 Hz, 1H, Ar-H), 6.37 (d, *J* = 2.7 Hz, 1H, Ar-H), 3.63 (s, 3H, -OMe), 2.19 (s, 2H, -CH_2), 2.13 (s, 2H, -CH_2), 0.99 (s, 6H, -CH_3). ^{13}C NMR (101 MHz, DMSO) δ 187.97, 156.16, 153.60, 129.46, 121.48, 116.64, 112.09, 111.42, 94.88, 55.24, 49.69, 41.25, 31.45, 27.61 ppm. HRMS (ESI) m/z calculated for $C_{15}H_{17}NO_2S$, $[M+H]^+$ 276.1058; found 276.1052.

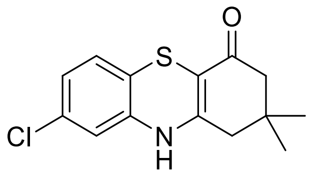

8-chloro-2,2-dimethyl-2,3-dihydro-1H-phenothiazin-4(10H)-one (3d)

Yield=65%. Yellow solid. M.p. 268.6-271.3℃. IR 3273, 2964, 2934, 1579, 1560, 1466, 1277, 860, 805 cm^{-1}. ^{1}H NMR (400 MHz, DMSO) δ 8.92 (s, 1H, N-H), 6.78 (dt, J = 14.4, 5.1 Hz, 2H, Ar-H), 6.56 (d, J = 1.9 Hz, 1H, Ar-H), 2.20 (s, 2H, $-CH_2$), 2.16 (s, 2H, $-CH_2$), 1.00 (s, 6H, -CH3). ^{13}C NMR (101 MHz, DMSO) δ 189.40, 154.00, 138.85, 131.22, 128.19, 124.36, 119.53, 115.54, 97.67, 50.13, 41.65, 32.03, 28.03 ppm. HRMS (ESI) m/z calculated for $C_{14}H_{14}ClNOS$, 279.0485; found 279.0482.

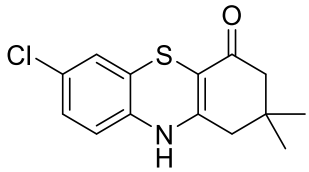

7-chloro-2,2-dimethyl-2,3-dihydro-1H-phenothiazin-4(10H)-one (3e)

Yield=81%. Brown solid. M.p. 268.6-271.3℃. IR 3272, 2958, 2908, 1579, 1467, 1380, 811, 664 cm^{-1}. ^{1}H NMR (400 MHz, DMSO) δ 8.96 (s, 1H, N-H), 6.97 – 6.77 (m, 2H, Ar-H), 6.52 (d, J = 8.4 Hz, 1H, Ar-H), 2.19 (s, 2H, $-CH_2$), 2.15 (s, 2H, $-CH_2$), 0.99 (s, 6H, -CH3). ^{13}C NMR (101 MHz, DMSO) δ 189.07, 154.23, 136.25, 128.25, 126.99, 126.10, 123.12, 117.28, 96.74, 50.11, 41.63, 31.98, 28.05 ppm. HRMS (ESI) m/z calculated for $C_{14}H_{14}ClNOS$, 279.0485; found 279.0497.

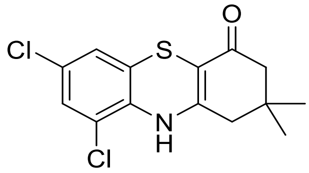

7,9-dichloro-2,2-dimethyl-2,3-dihydro-1H-phenothiazin-4(10H)-one (3f)

Yield=55%. Yellow solid. M.p. 193.2-195.1℃. IR 3270, 2954, 1588, 1486, 1266, 807, 638 cm^{-1}. ^{1}H NMR (400 MHz, DMSO) δ 7.98 (s, 1H, N-H), 7.18 (d, J = 2.3 Hz, 1H, Ar-H), 6.92 (d, J = 2.2 Hz, 1H, Ar-H), 2.42 (s, 2H, $-CH_2$), 2.19 (s, 2H, $-CH_2$), 0.99 (s, 6H, -CH3). ^{13}C NMR (101 MHz, DMSO) δ 190.14, 154.62, 133.38, 128.32, 127.27, 125.28, 125.23, 120.60, 99.15, 50.09, 41.46, 31.99, 28.00 ppm. HRMS (ESI) m/z calculated for $C_{14}H_{13}Cl_2NOS$, $[M+H]^+$ 314.0173; found 314.0179.

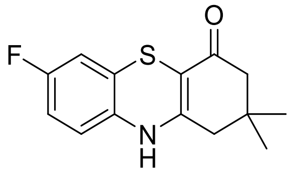

7-fluoro-2,2-dimethyl-2,3-dihydro-1H-phenothiazin-4(10H)-one (3g)

Yield=85%. Yellow solid. M.p. 268.6-271.3℃. IR 3274, 2956, 1593, 1476, 1344, 850, 807, 689 cm^{-1}. ^{1}H NMR (400 MHz, DMSO) δ 8.92 (s, 1H, N-H), 6.74 – 6.65 (m, 2H, Ar-H), 6.54 (dd, J = 9.4, 5.2 Hz, 1H, Ar-H), 2.19 (s, 2H, $-CH_2$), 2.15 (s, 2H, $-CH_2$), 1.00 (s, 6H, -CH3). ^{13}C NMR (101 MHz, DMSO) δ 188.91, 160.41, 158.01, 154.42, 133.55,

133.52, 123.08, 123.00, 117.22, 117.13, 113.99, 113.74, 113.46, 113.24, 95.73, 50.13, 41.66, 31.96, 28.06 ppm. HRMS (ESI) m/z calculated for $C_{14}H_{14}FNOS$, $[M+H]^+$ 264.0858; found 264.0864.

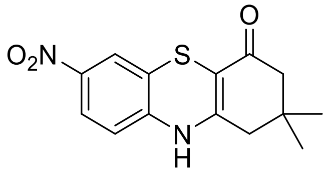

2,2-dimethyl-7-nitro-2,3-dihydro-1H-phenothiazin-4(10H)-one (3h)
Yield=92%. Purple solid. M.p. >300℃. IR 3270, 2966, 1590, 1473, 1332, 878, 714, 654 cm^{-1}. ^{1}H NMR (400 MHz, DMSO) δ 9.34 (s, 1H, N-H), 7.72 (dd, *J* = 8.7, 2.4 Hz, 1H, Ar-H), 7.52 (d, *J* = 2.2 Hz, 1H, Ar-H), 6.59 (d, *J* = 8.8 Hz, 1H, Ar-H), 2.19 (d, *J* = 3.9 Hz, 4H, $-CH_2$), 1.00 (s, 6H, -CH3). ^{13}C NMR (101 MHz, DMSO) δ 189.98, 152.99, 144.05, 143.67, 124.41, 122.38, 121.68, 115.55, 98.95, 50.04, 41.40, 32.06, 28.00 ppm. HRMS (ESI) m/z calculated for $C_{14}H_{14}N_2O_3S$, $[M+H]^+$ 291.0803; found 291.0799.

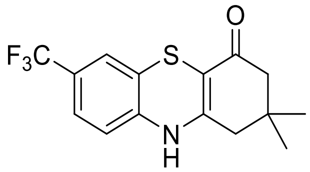

2,2-dimethyl-7-(trifluoromethyl)-2,3-dihydro-1H-phenothiazin-4(10H)-one (3i)
Yield=94%. Yellow solid. M.p. >300℃. IR 3260, 2958, 1596, 1569, 1479, 1305, 830, 704 cm^{-1}. ^{1}H NMR (400 MHz, DMSO) δ 9.11 (s, 1H, N-H), 7.20 (d, *J* = 7.9 Hz, 1H, Ar-H), 7.06 (s, 1H, Ar-H), 6.64 (d, *J* = 8.2 Hz, 1H, Ar-H), 2.21 (s, 2H, $-CH_2$), 2.18 (s, 2H, $-CH_2$), 1.01 (s, 6H, -CH3). ^{13}C NMR (101 MHz, DMSO) δ 189.55, 154.03, 141.17, 125.55, 125.28, 124.96, 124.91, 124.87, 123.53, 123.50, 122.85, 122.14, 115.93, 97.93, 50.10, 41.55, 32.02, 28.01 ppm. HRMS (ESI) m/z calculated for $C_{15}H_{14}F_3NOS$, $[M+H]^+$ 314.0826; found 314.0833.

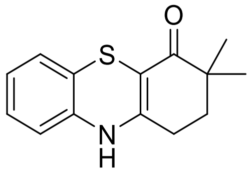

3,3-dimethyl-2,3-dihydro-1H-phenothiazin-4(10H)-one (3j)
Yield=96%. Yellow solid. M.p. 278.4-280.3℃. IR 3253, 2972, 1610, 1581, 1471, 1238, 747 cm^{-1}. ^{1}H NMR (400 MHz, DMSO) δ 8.87 (s, 1H, N-H), 6.86 (ddd, *J* = 8.0, 6.2, 3.6 Hz, 1H, Ar-H), 6.74 (dd, *J* = 7.4, 5.5 Hz, 2H, Ar-H), 6.55 (d, *J* = 7.8 Hz, 1H, Ar-H), 2.36 (t, *J* = 6.2 Hz, 2H, $-CH_2$), 1.69 (t, *J* = 6.2 Hz, 2H, $-CH_2$), 1.02 (s, 6H, -CH3). ^{13}C NMR (101 MHz, DMSO) δ 194.31, 154.87, 137.04, 127.28, 126.87, 124.85, 120.36, 115.99, 97.12, 34.06, 25.49, 25.29 ppm. HRMS (ESI) m/z calculated for $C_{14}H_{15}NOS$, $[M+H]^+$ 246.0953; found 246.0957.

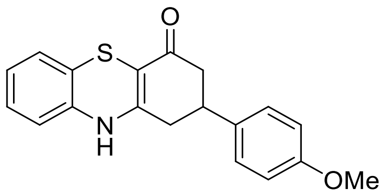

2-(4-methoxyphenyl)-2,3-dihydro-1H-phenothiazin-4(10H)-one (3k)
Yield=72%. Yellow solid. M.p. 285.8-287.3℃. IR 3245, 2941, 1580, 1565, 1468, 1248, 823, 752 cm^{-1}. ^{1}H NMR (400 MHz, DMSO) δ 8.97 (s, 1H, N-H), 7.24 (d, *J* = 7.9 Hz, 2H, Ar-H), 6.89 (d, *J* = 7.9 Hz, 3H, Ar-H), 6.75 (d, *J* = 2.8 Hz, 2H, Ar-H), 6.55 (d, *J* = 7.5 Hz, 1H, Ar-H), 3.73 (s, 3H, -CH3), 2.76 – 2.28 (m, 5H, $-CH_2$, -CH). ^{13}C NMR (101 MHz, DMSO) δ 188.86, 158.46, 155.51, 137.09, 135.58, 128.34, 127.41, 126.96, 125.10, 120.32, 116.21, 114.34, 98.19, 55.49, 43.88, 37.35, 35.85 ppm. HRMS (ESI) m/z calculated for $C_{19}H_{17}NO_2S$, $[M+H]^+$ 324.1058; found 324.1042.

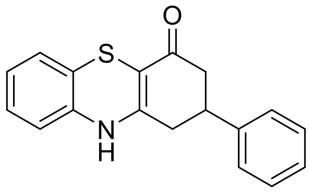

2-phenyl-2,3-dihydro-1H-phenothiazin-4(10H)-one (3l)
Yield=60%. Red solid. M.p. 241.2-243.1℃. IR 3253, 2954, 1582, 1565, 1470, 1246, 750, 699 cm^{-1}. ^{1}H NMR (400 MHz, DMSO) δ 8.99 (s, 1H, N-H), 7.34 (d, *J* = 4.1 Hz, 4H, Ar-H), 7.25 (dd, *J* = 8.3, 4.1 Hz, 1H, Ar-H), 6.94 – 6.83 (m, 1H, Ar-H), 6.76 (d, *J* = 4.0 Hz, 2H, Ar-H), 6.57 (s, 1H, Ar-H), 3.31 (dd, *J* = 20.3, 8.7 Hz, 1H, -CH), 2.74 – 2.57 (m, 2H, $-CH_2$), 2.53 – 2.43 (m, 2H, $-CH_2$). ^{13}C NMR (101 MHz, DMSO) δ 188.71, 155.46, 143.62, 137.07, 128.99, 127.42, 127.36, 127.18, 126.96, 125.12, 120.32, 116.24, 98.21, 43.59, 38.10, 35.55 ppm. HRMS (ESI) m/z calculated for $C_{18}H_{15}NOS$, $[M+H]^+$ 294.0953; found 294.0961.

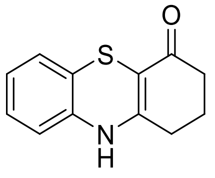

2,3-dihydro-1H-phenothiazin-4(10H)-one (3m)
Yield=47%. Yellow solid. M.p. 199.5-201.7℃. IR 3263, 2933, 1585, 1508, 1466, 1296, 737, 686 cm^{-1}. ^{1}H NMR (400 MHz, DMSO) δ 8.92 (s, 1H, N-H), 6.91 – 6.81 (m, 1H, Ar-H), 6.81 – 6.66 (m, 2H, Ar-H), 6.55 (d, *J* = 7.8 Hz, 1H, Ar-H), 2.33 (t, *J* = 6.1 Hz, 2H, $-CH_2$), 2.25 (t, *J* = 6.4 Hz, 2H, $-CH_2$), 1.89 – 1.75 (m, 2H, $-CH_2$). ^{13}C NMR (101 MHz, DMSO) δ 189.48, 156.47, 137.10, 127.32, 126.88, 124.95, 120.40, 116.11, 98.33, 36.61, 28.45, 20.60 ppm. HRMS (ESI) m/z calculated for $C_{12}H_{11}NOS$, $[M+H]^+$ 218.0640; found 218.0641.

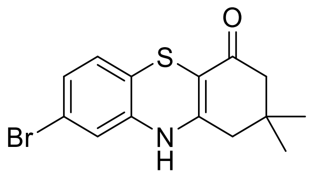

8-bromo-2,2-dimethyl-2,3-dihydro-1H-phenothiazin-4(10H)-one (3o)

Yield=80%. Yellow solid. M.p. 231.7-232-4℃. IR 3263, 2954, 1647, 1558, 1464, 1268, 877, 805 cm^{-1}. ^{1}H NMR (400 MHz, DMSO) δ 8.90 (s, 1H, N-H), 6.90 (dd, *J* = 8.2, 1.6 Hz, 1H, Ar-H), 6.72 – 6.63 (m, 2H, Ar-H), 2.18 (s, 2H, $-CH_2$), 2.15 (s, 2H, $-CH_2$), 0.99 (s, 6H, -CH3). ^{13}C NMR (101 MHz, DMSO) δ 188.88, 153.57, 138.54, 127.99, 126.78, 119.61, 118.70, 117.79, 97.14, 49.64, 41.16, 31.54, 27.54 ppm. HRMS (ESI) m/z calculated for $C_{14}H_{14}BrNOS$, $[M+H]^+$ 324.0058; found 324.0059.

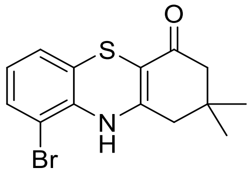

9-bromo-2,2-dimethyl-2,3-dihydro-1H-phenothiazin-4(10H)-one (3p)

Yield=68%. Yellow solid. M.p. >300℃. IR 3277, 2957, 1574, 1503, 1441, 1294, 763, 716 cm^{-1}. ^{1}H NMR (400 MHz, DMSO) δ 7.57 (s, 1H, N-H), 7.18 (dd, *J* = 8.0, 1.3 Hz, 1H, Ar-H), 6.83 – 6.77 (m, 1H, Ar-H), 6.70 (t, *J* = 7.8 Hz, 1H, Ar-H), 2.44 (s, 2H, -CH_2), 2.19 (s, 2H, $-CH_2$), 0.99 (s, 6H, -CH3). ^{13}C NMR (101 MHz, DMSO) δ 190.16, 155.01, 135.19, 131.41, 126.61, 126.12, 123.01, 110.32, 99.77, 50.24, 41.64, 32.01, 28.00 ppm. HRMS (ESI) m/z calculated for $C_{14}H_{14}BrNOS$, $[M+H]^+$ 324.0058; found 324.0059.

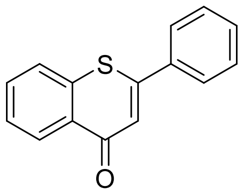

2-phenyl-4H-thiochromen-4-one (5a)

Yield=51%. Red solid. M.p. 105.5-106.9℃. IR 2923, 1618, 1587, 1435, 1332, 1099, 863, 758, 730, 695 cm^{-1}. ^{1}H NMR (400 MHz, Chloroform-*d*) δ 8.54 (d, *J* = 7.8 Hz, 1H, Ar-H), 7.70 – 7.44 (m, 8H, Ar-H), 7.24 (s, 1H, -CH). ^{13}C NMR (101 MHz, $CDCl_3$) δ 180.33, 152.84, 137.27, 136.05, 131.17, 130.38, 130.34, 128.82, 128.13, 127.34, 126.51, 126.02, 122.88, 122.85 ppm. HRMS (ESI) m/z calculated for $C_{15}H_{10}OS$, $[M+H]^+$ 239.0531; found 239.0529.

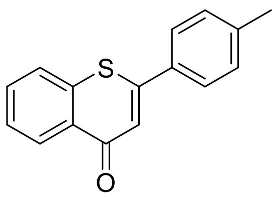

2-p-tolyl-4H-thiochromen-4-one (5b)

Yield=53%. Red solid. M.p. 106.7-108.3℃. IR 3028, 1604, 1587, 1507, 1431, 1336, 1131, 1103, 878, 775, 687 cm^{-1}. ^1H NMR (400 MHz, Chloroform-*d*) δ 8.55 (dd, *J* = 8.0, 1.4 Hz, 1H, Ar-H), 7.69 – 7.52 (m, 5H, Ar-H), 7.30 (d, *J* = 7.9 Hz, 2H, Ar-H), 7.27 (s, 1H, -CH), 2.43 (s, 3H, $-CH_3$). ^{13}C NMR (101 MHz, $CDCl_3$) δ 180.38, 152.95, 152.92, 140.93, 137.31, 133.18, 131.10, 130.35, 129.52, 128.10, 127.26, 126.34, 125.99, 122.25, 20.91 ppm. HRMS (ESI) m/z calculated for $C_{16}H_{12}OS$, $[M+H]^+$ 253.0687; found 253.0685.

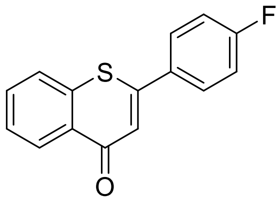

2-(4-fluorophenyl)-4H-thiochromen-4-one (5c)

Yield=50%. Red solid. M.p. 152.8-153.9℃. IR 1617, 1586, 1502, 1441, 1335, 1228, 1106, 1100, 847, 779, 743, 686 cm^{-1}. ^1H NMR (400 MHz, Chloroform-*d*) δ 8.55 (dd, *J* = 7.8, 1.3 Hz, 1H, Ar-H), 7.74 – 7.54 (m, 5H, Ar-H), 7.26 – 7.16 (m, 3H, Ar-H). ^{13}C NMR (101 MHz, $CDCl_3$) δ 180.23, 165.13, 162.62, 151.70, 137.04, 132.20, 132.17, 131.30, 130.19, 128.57, 128.49, 128.17, 127.48, 125.98, 122.81, 116.12, 115.90 ppm. HRMS (ESI) m/z calculated for $C_{15}H_9FOS$, $[M+H]^+$ 257.0436; found 257.0442.

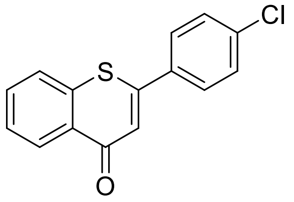

2-(4-chlorophenyl)-4H-thiochromen-4-one (5d)

Yield=51%. Red solid. M.p. 160.8-162.6℃. IR 2161, 2028, 1629, 1589, 1485, 1327, 1088, 1010, 900, 828, 776, 707, 684 cm^{-1}. ^1H NMR (400 MHz, Chloroform-*d*) δ 8.55 (d, *J* = 7.9 Hz, 1H, Ar-H), 7.71 – 7.52 (m, 5H, Ar-H), 7.48 (d, *J* = 8.1 Hz, 2H, Ar-H), 7.24 (s, 1H, -CH). ^{13}C NMR (101 MHz, $CDCl_3$) δ 180.21, 151.38, 136.94, 136.71, 134.46, 131.33, 130.26, 129.11, 128.19, 127.77, 127.52, 126.03, 123.00 ppm. HRMS (ESI) m/z calculated for $C_{15}H_9ClOS$, $[M+H]^+$ 273.0141; found 273.0147.

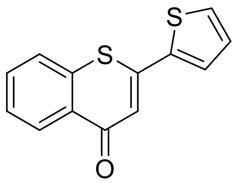

2-(thiophen-2-yl)-4H-thiochromen-4-one (5e)

Yield=60%. Red solid. M.p. 97.8-99.1℃. IR 2161, 1610, 1585, 1544, 1417, 1321, 1228, 1098, 848, 825, 771, 718 cm^{-1}. ^{1}H NMR (400 MHz, Chloroform-*d*) δ 8.50 (dt, *J* = 8.0, 1.1 Hz, 1H, Ar-H), 7.63 – 7.59 (m, 2H, Ar-H), 7.56 – 7.50 (m, 3H, Ar-H), 7.27 (s, 1H, -CH), 7.15 (dd, *J* = 5.1, 3.7 Hz, 1H, Ar-H). ^{13}C NMR (101 MHz, $CDCl_3$) δ 180.10, 145.26, 138.61, 136.36, 136.29, 131.29, 130.41, 128.81, 128.08, 128.06, 127.34, 126.88, 125.75, 120.82 ppm. HRMS (ESI) m/z calculated for $C_{13}H_8OS_2$, $[M+H]^+$ 245.0095; found 245.0105.

2,2-dimethyl-2,3-dihydro-1H-phenothiazin-4(10H)-one (3a)

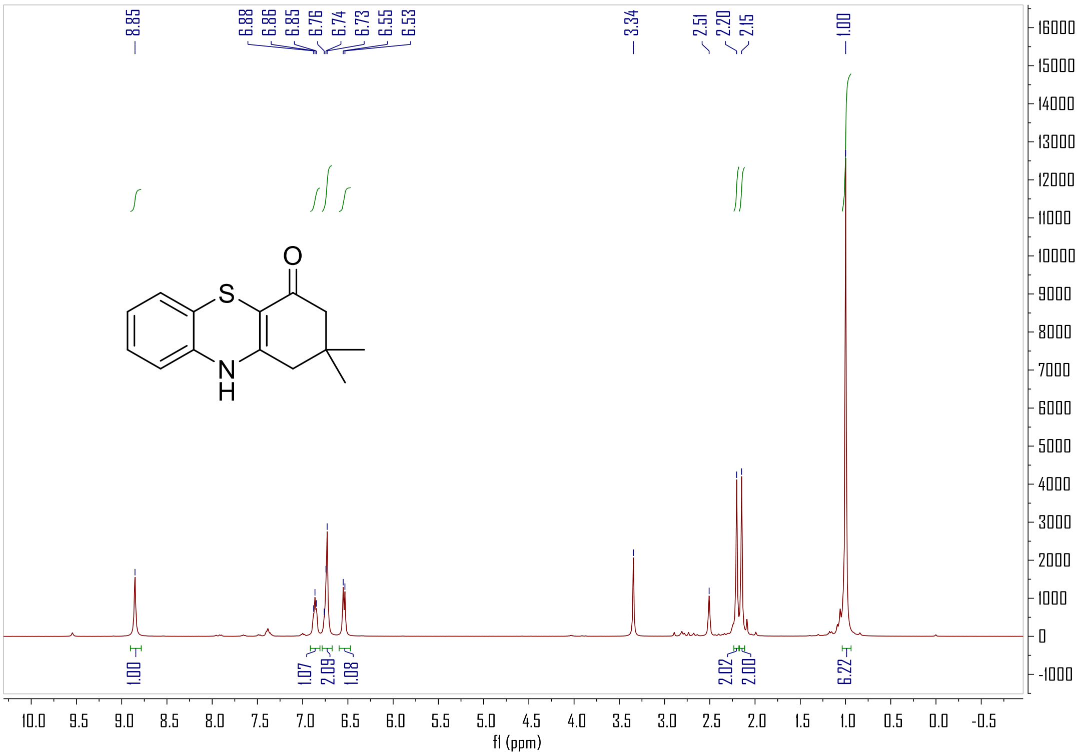

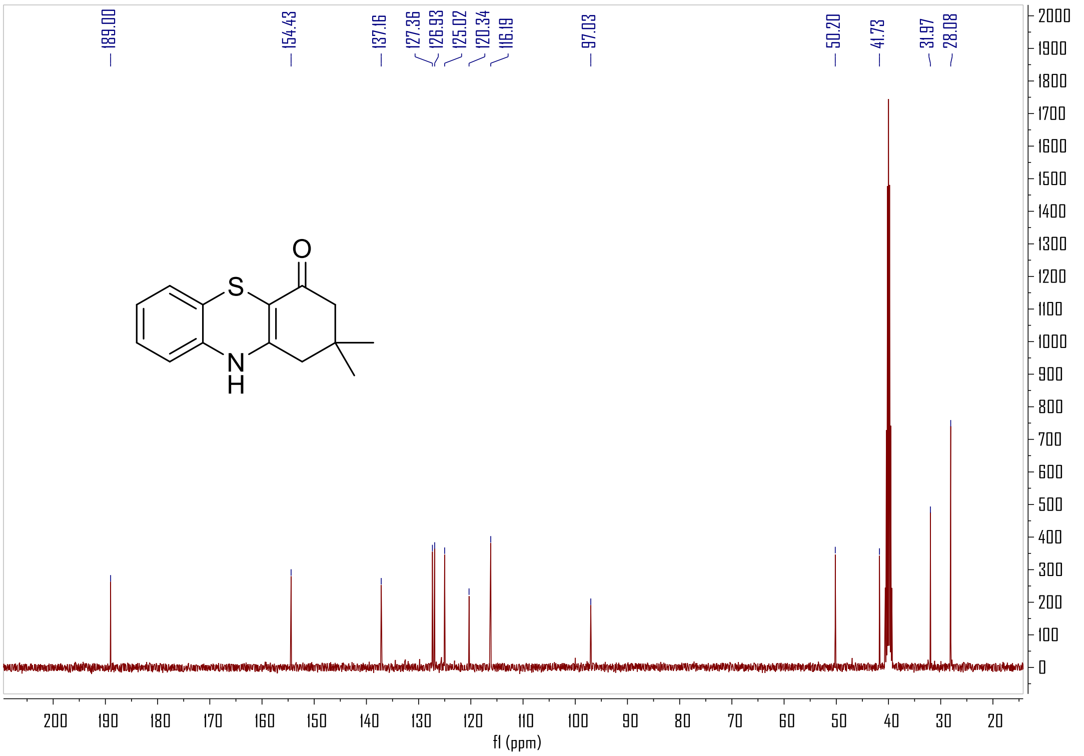

2,2,7-trimethyl-2,3-dihydro-1H-phenothiazin-4(10H)-one (3b)

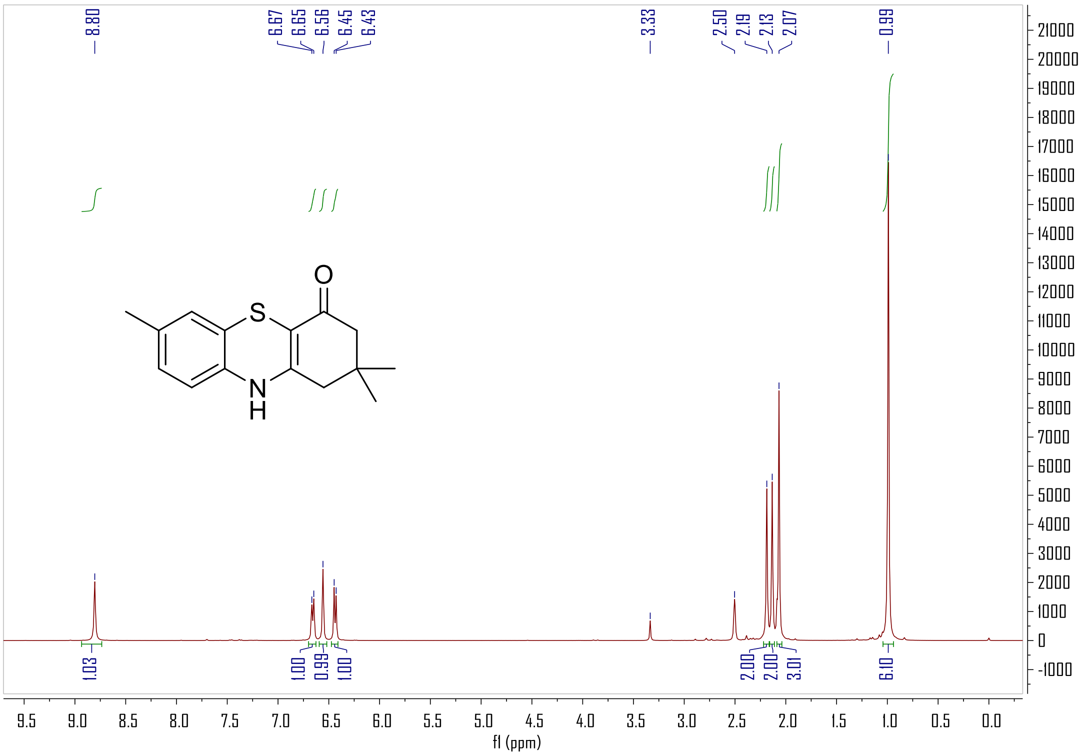

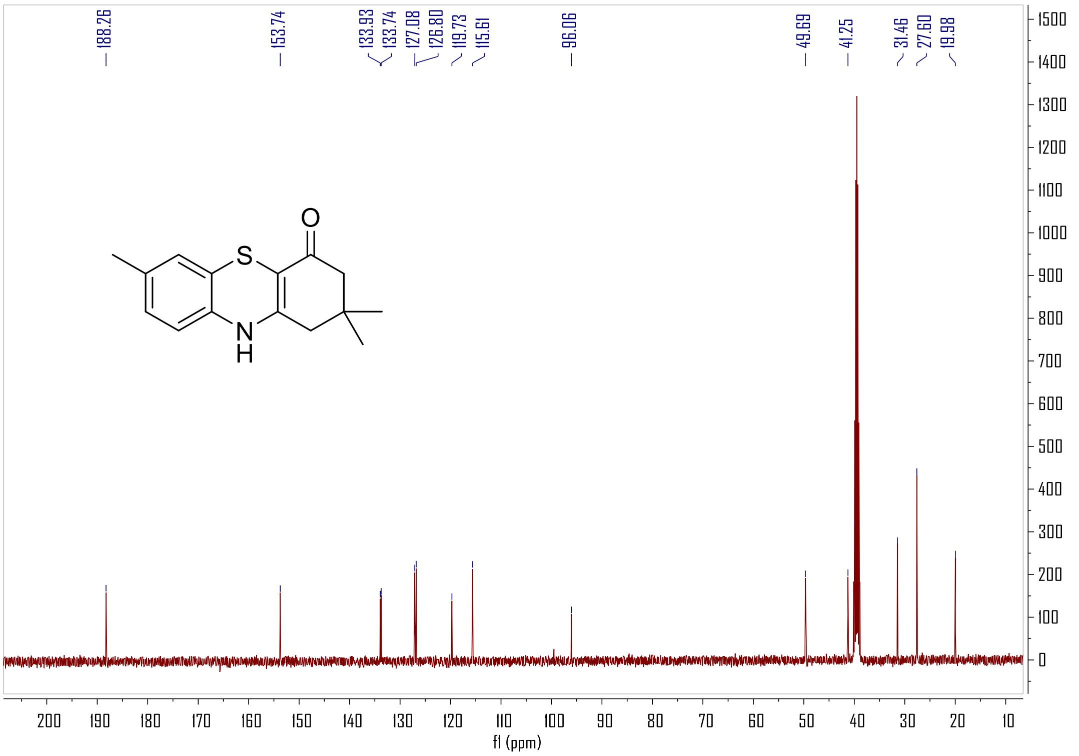

7-methoxy-2,2-dimethyl-2,3-dihydro-1H-phenothiazin-4(10H)-one (3c)

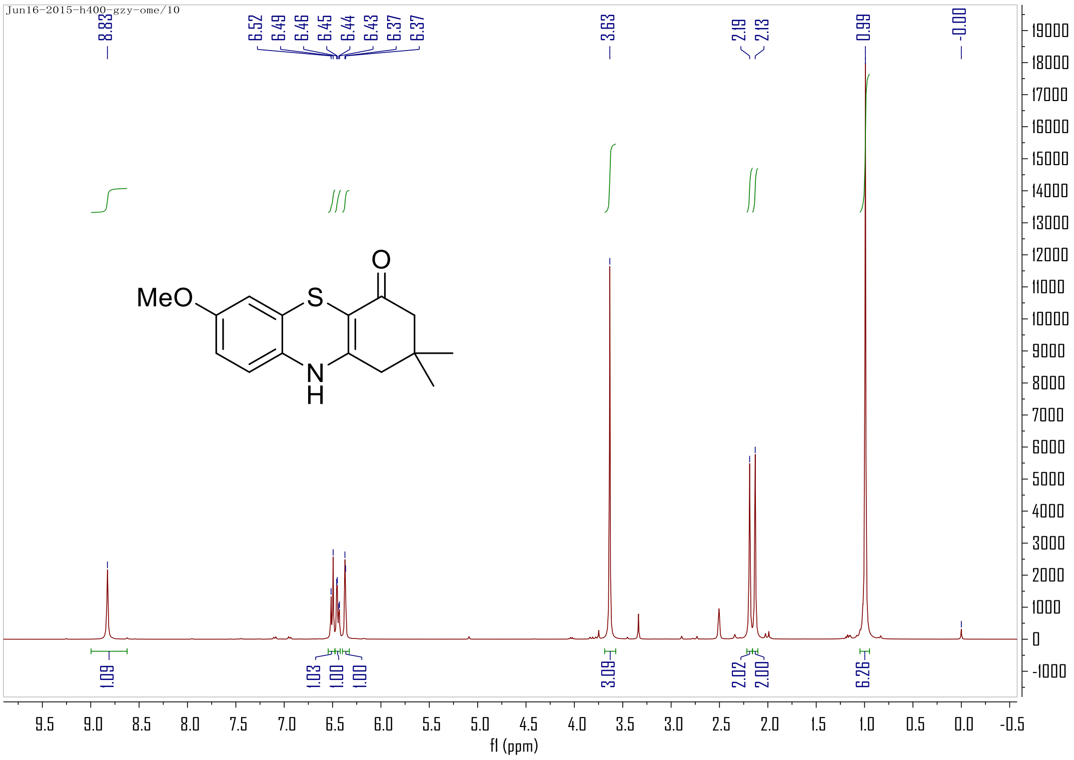

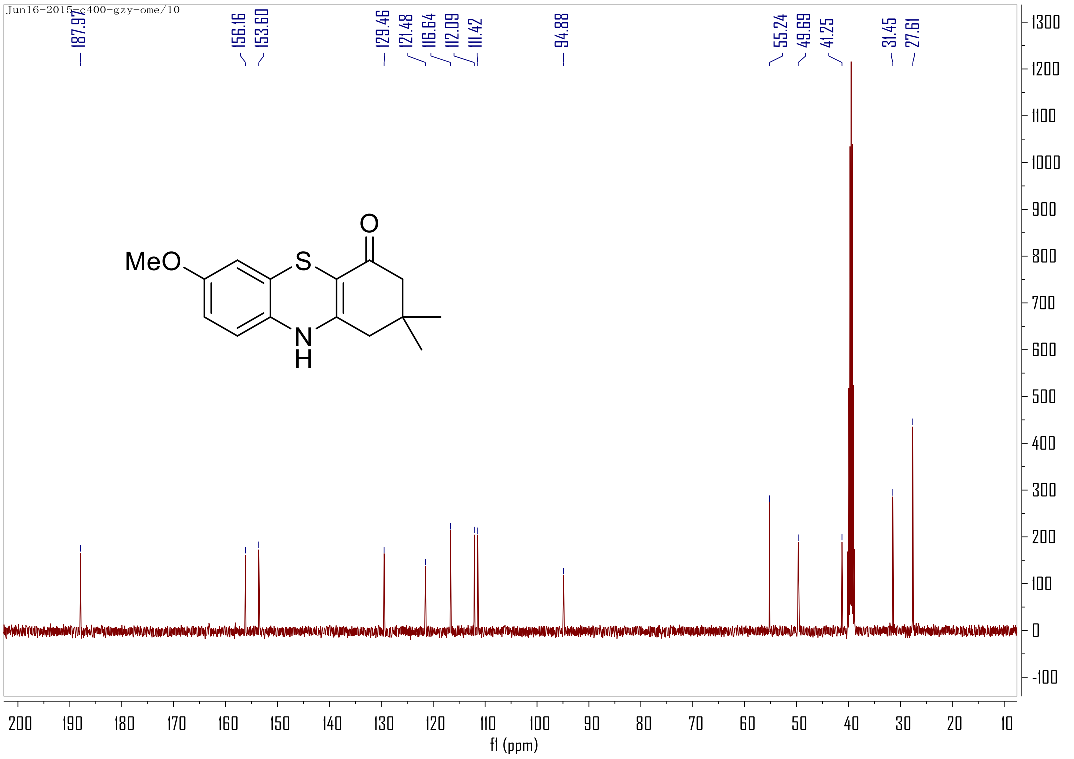

8-chloro-2,2-dimethyl-2,3-dihydro-1H-phenothiazin-4(10H)-one (3d)

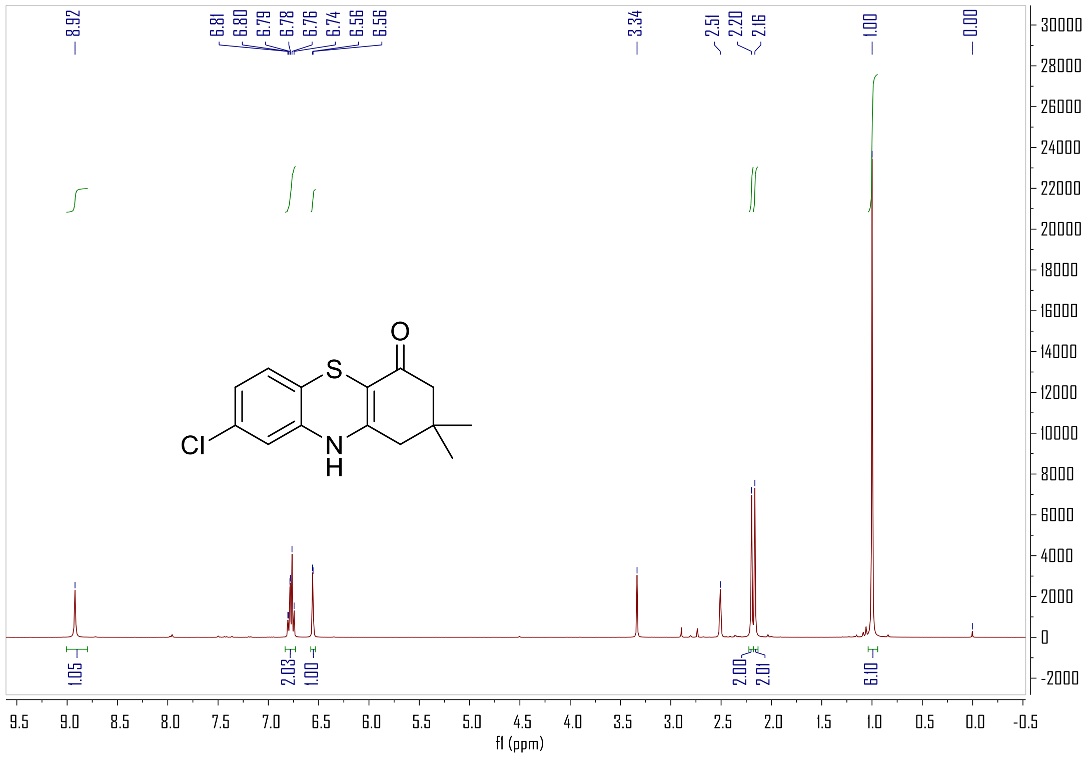

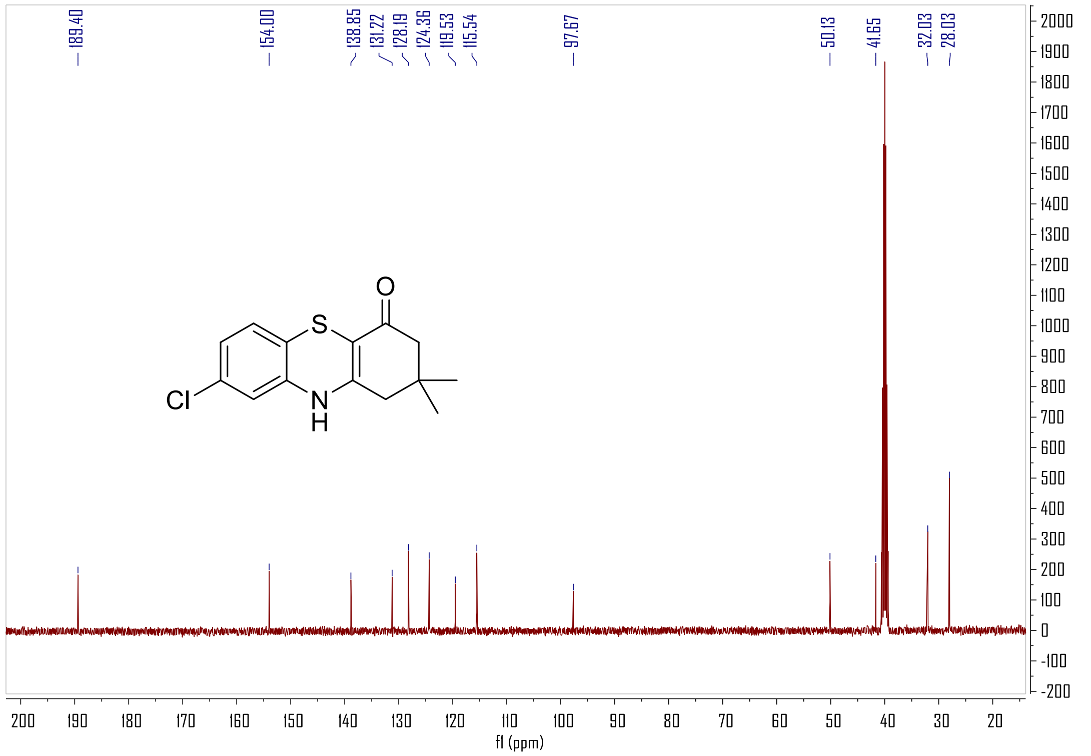

7-chloro-2,2-dimethyl-2,3-dihydro-1H-phenothiazin-4(10H)-one (3e)

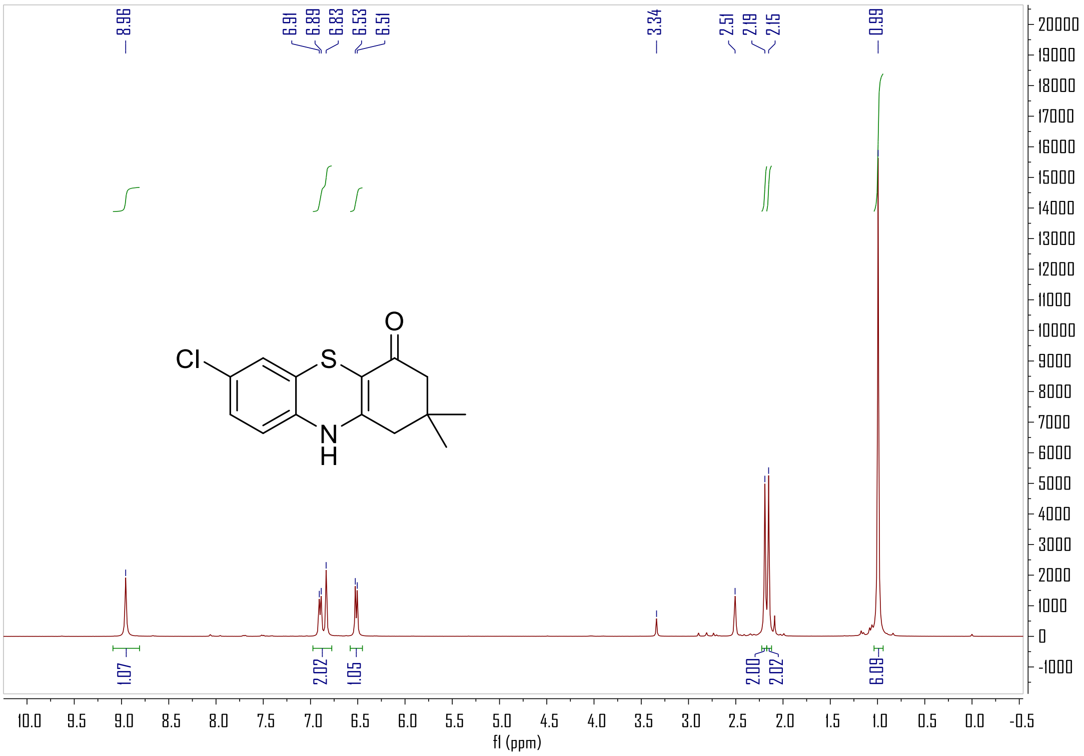

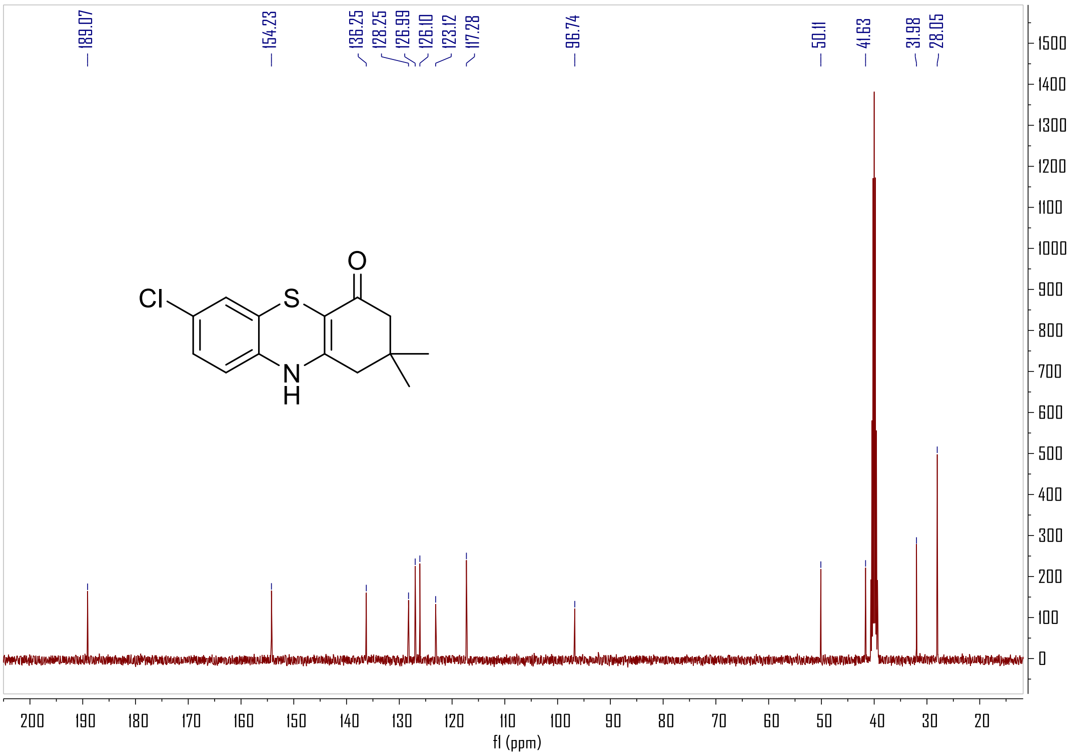

7,9-dichloro-2,2-dimethyl-2,3-dihydro-1H-phenothiazin-4(10H)-one (3f)

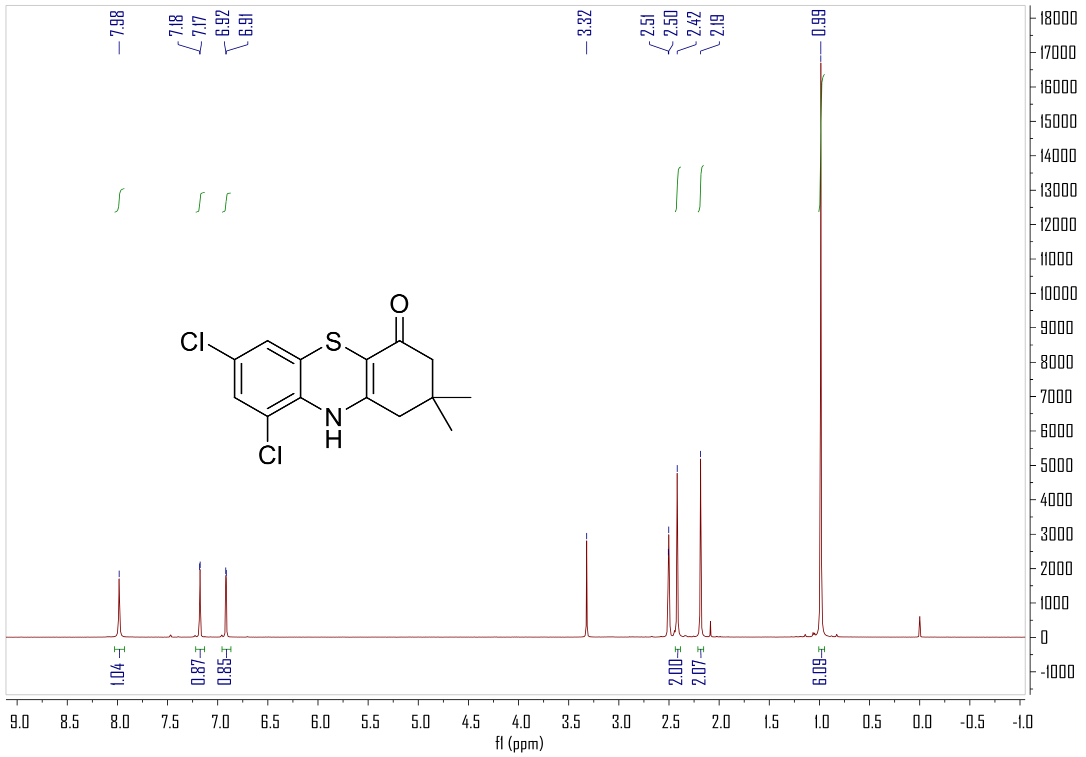

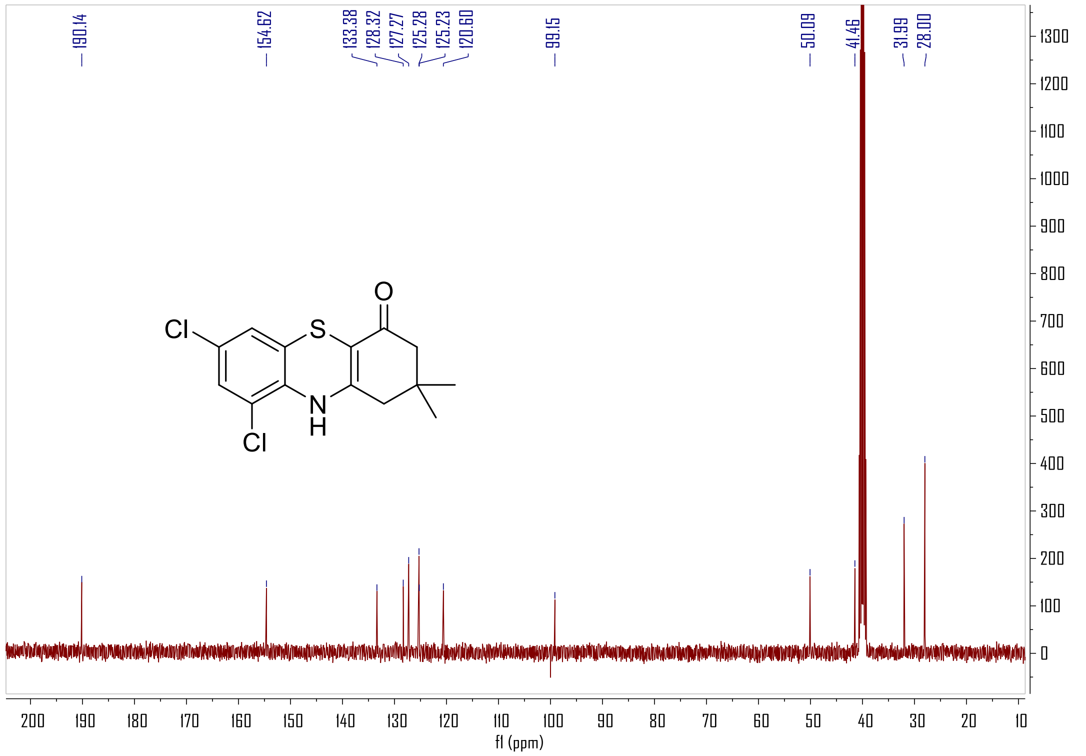

7-fluoro-2,2-dimethyl-2,3-dihydro-1H-phenothiazin-4(10H)-one (3g)

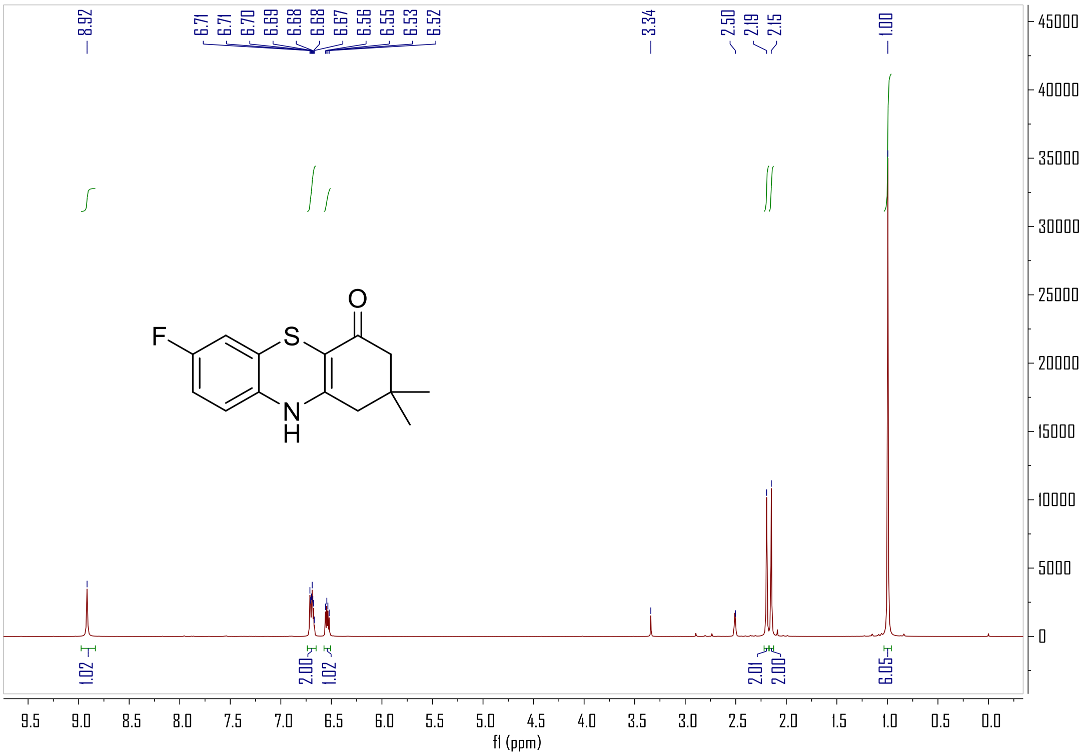

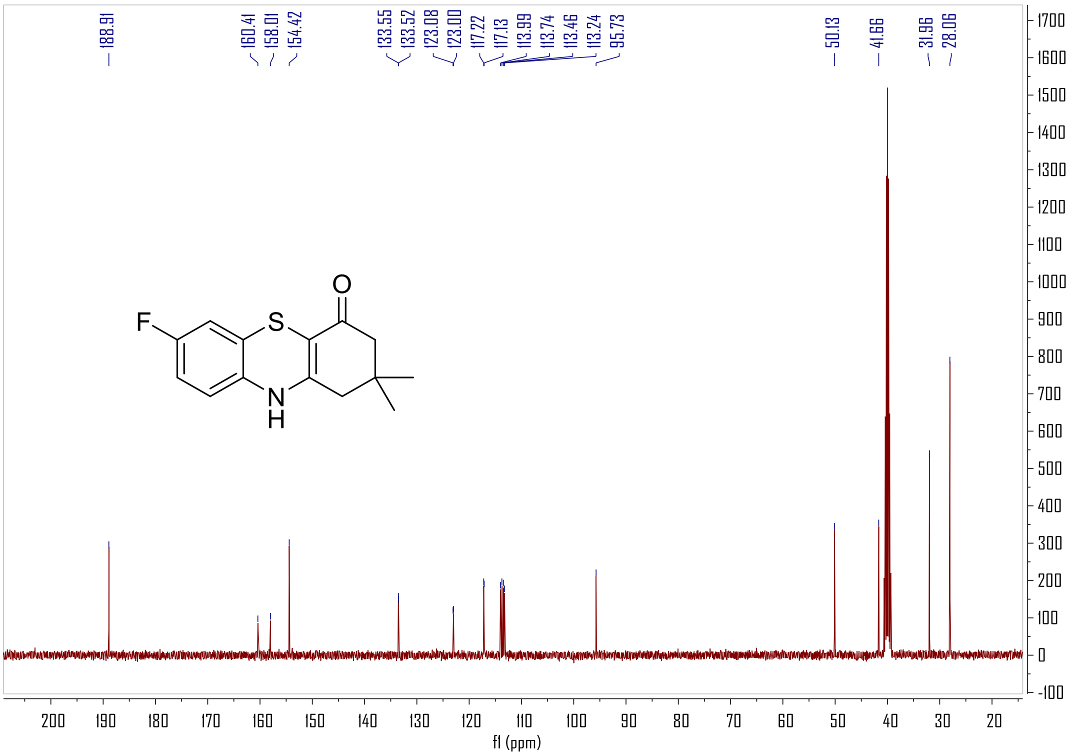

2,2-dimethyl-7-nitro-2,3-dihydro-1H-phenothiazin-4(10H)-one (3h)

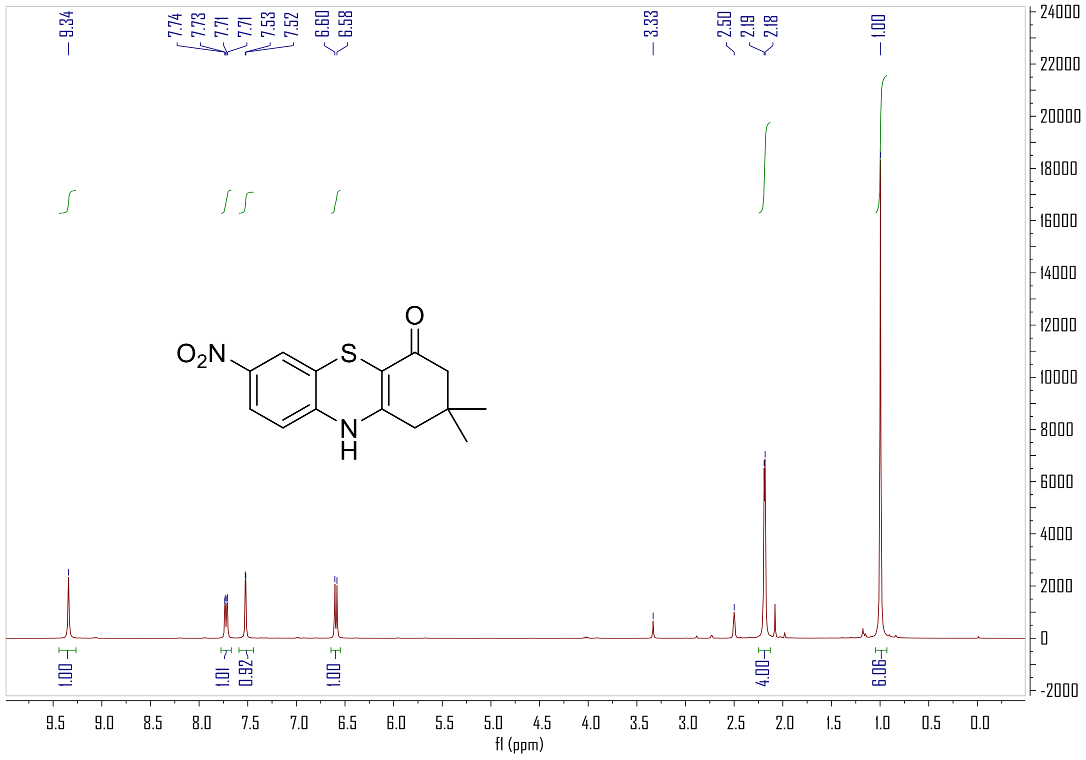

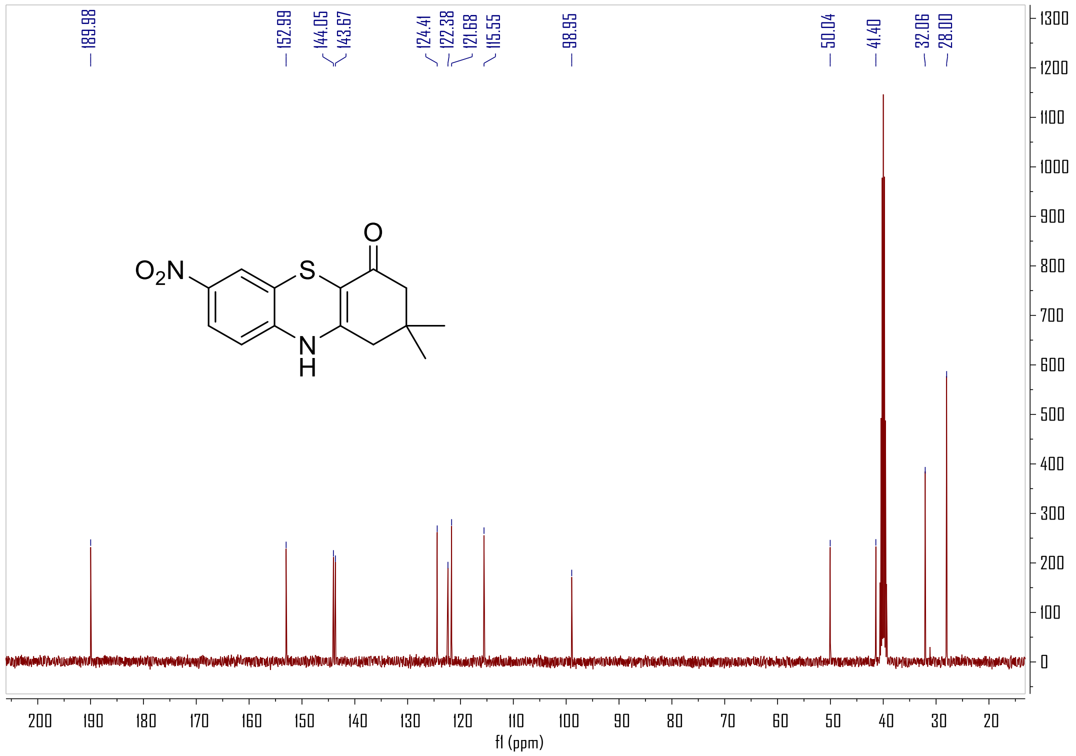

2,2-dimethyl-7-(trifluoromethyl)-2,3-dihydro-1H-phenothiazin-4(10H)-one (3i)

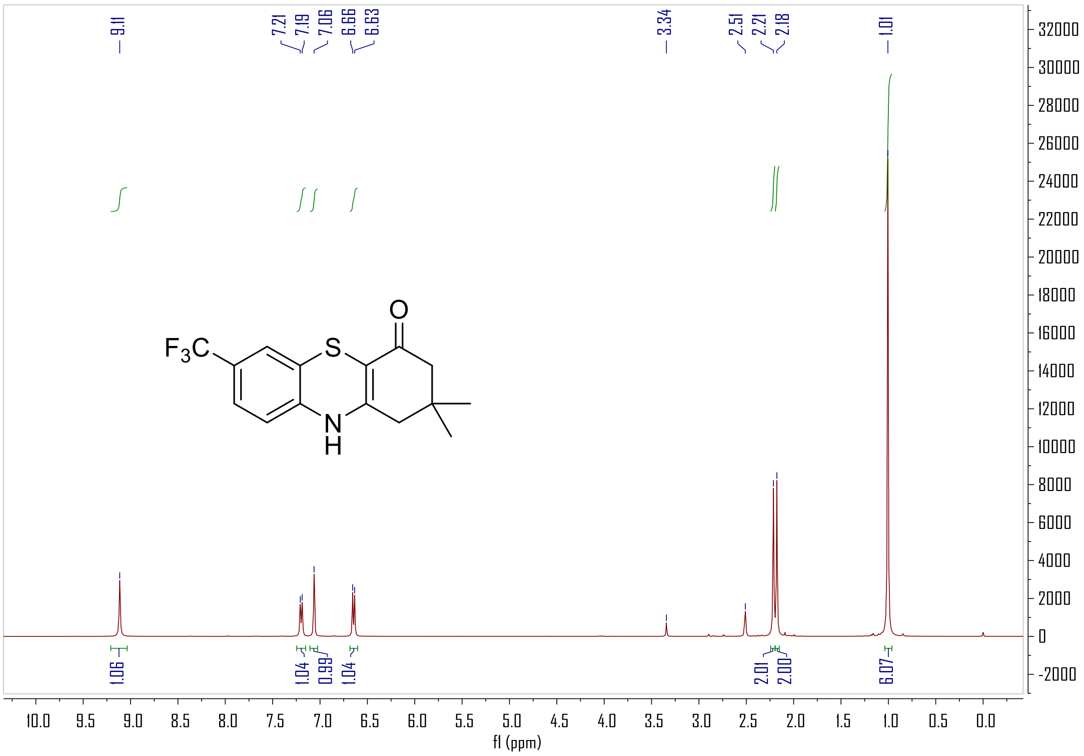

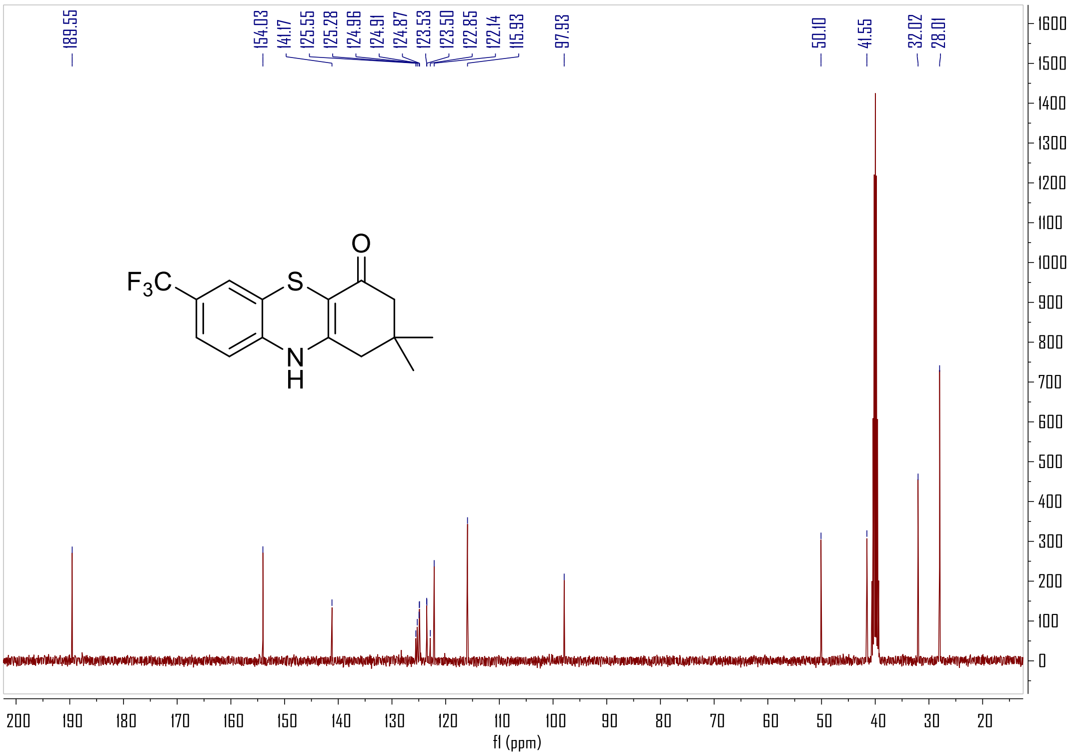

3,3-dimethyl-2,3-dihydro-1H-phenothiazin-4(10H)-one (3j)

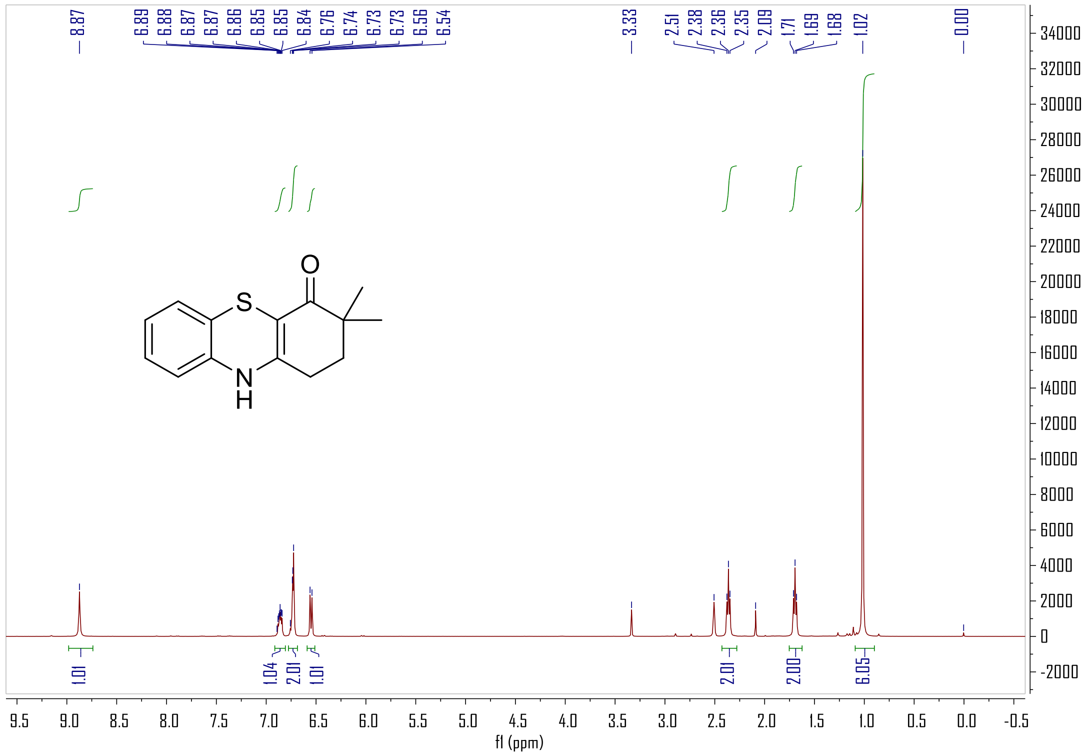

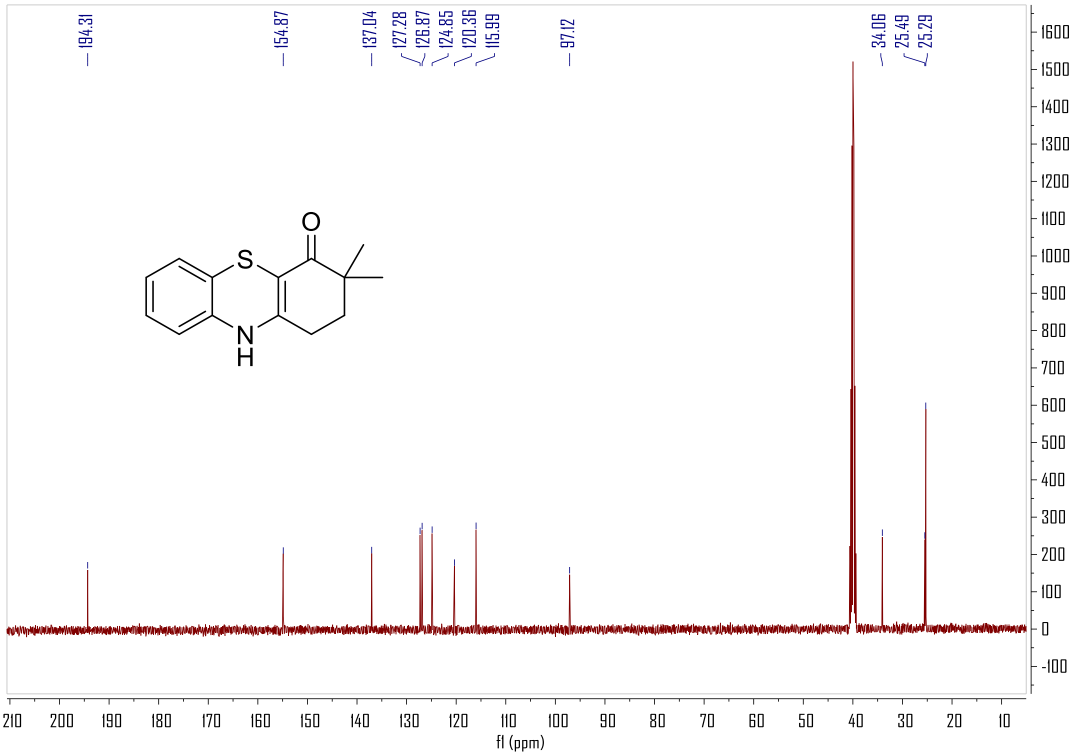

2-(4-methoxyphenyl)-2,3-dihydro-1H-phenothiazin-4(10H)-one (3k)

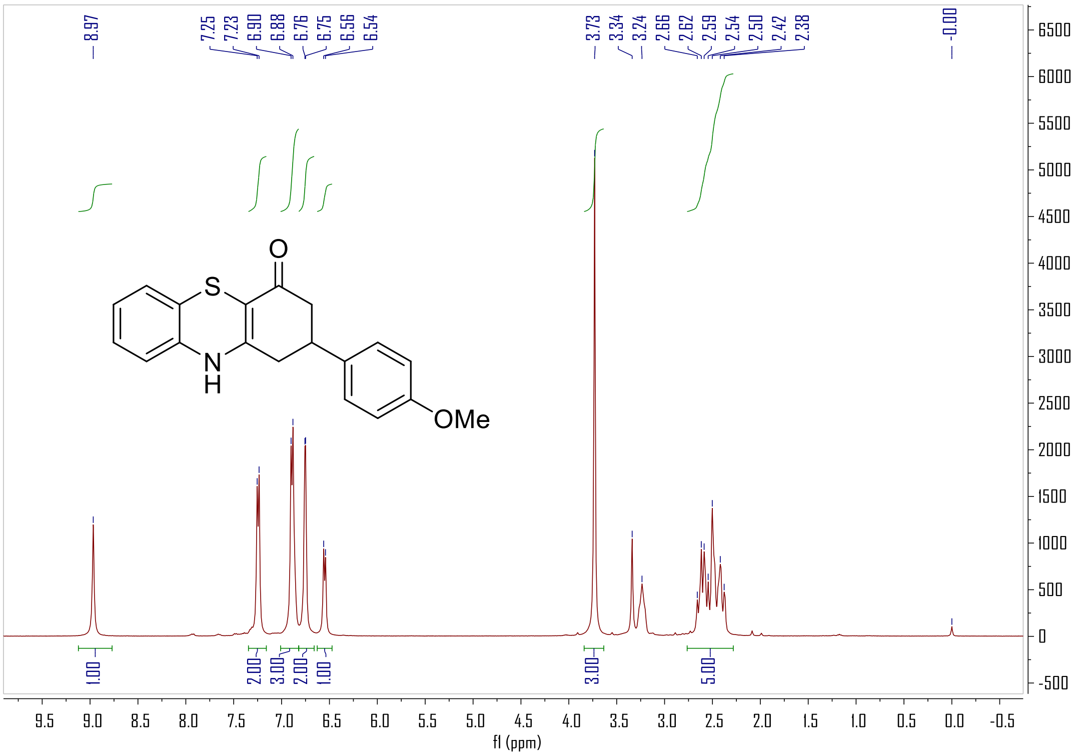

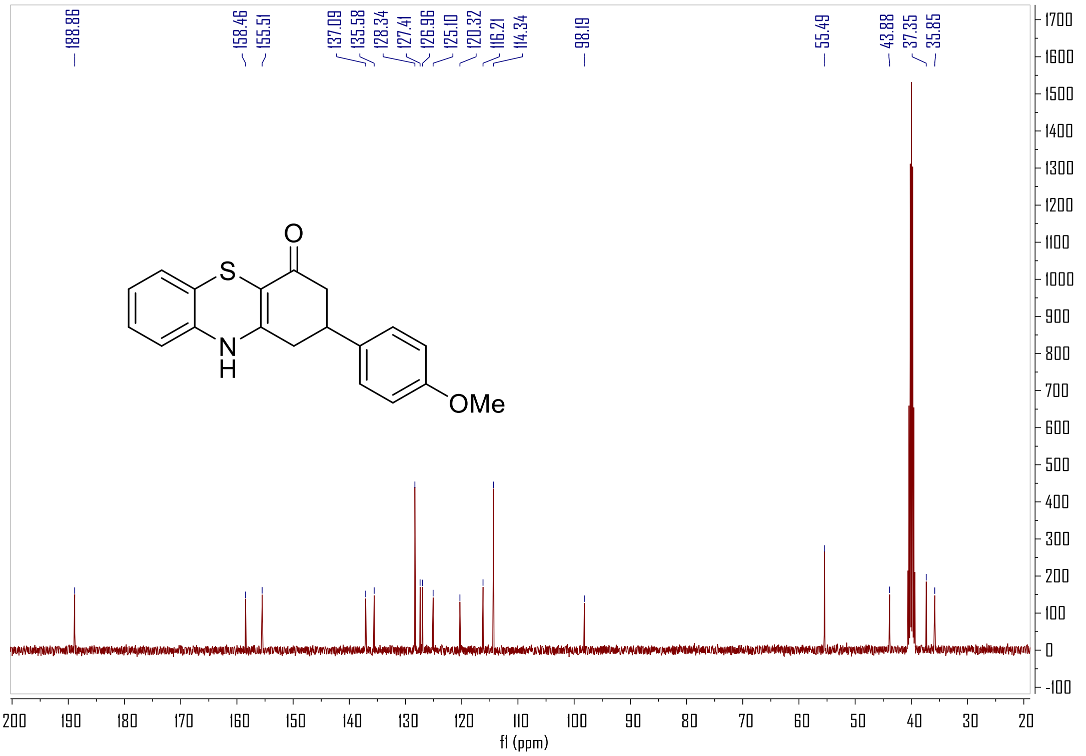

2-phenyl-2,3-dihydro-1H-phenothiazin-4(10H)-one (3l)

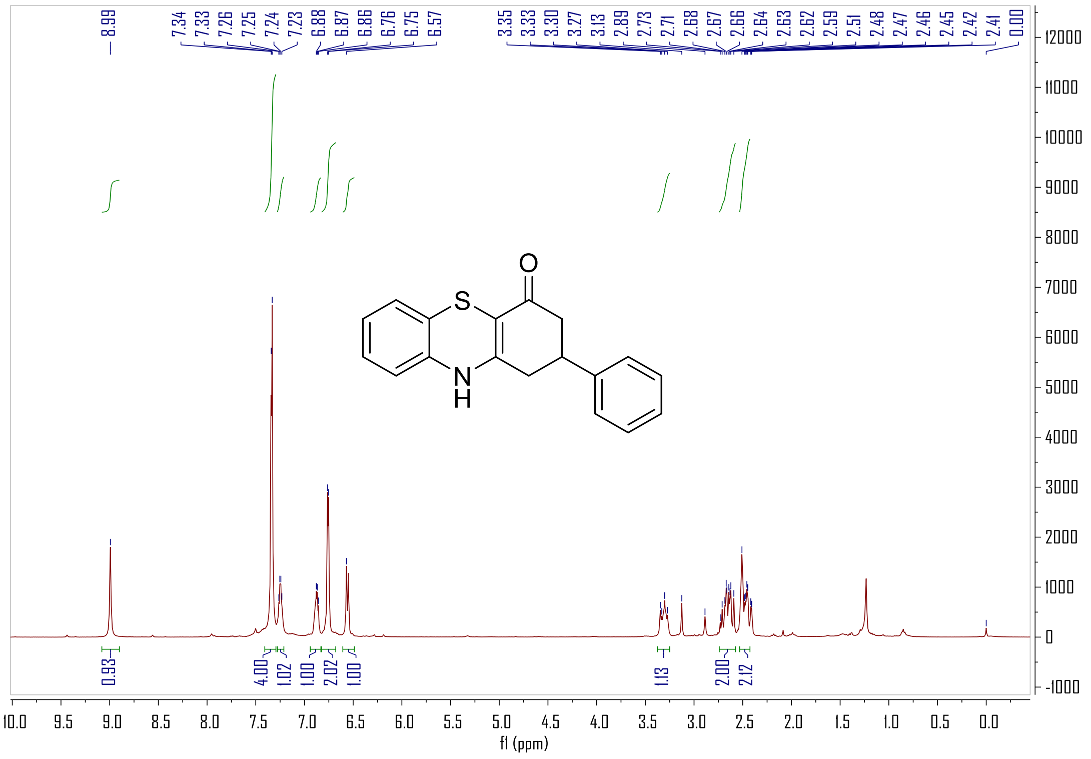

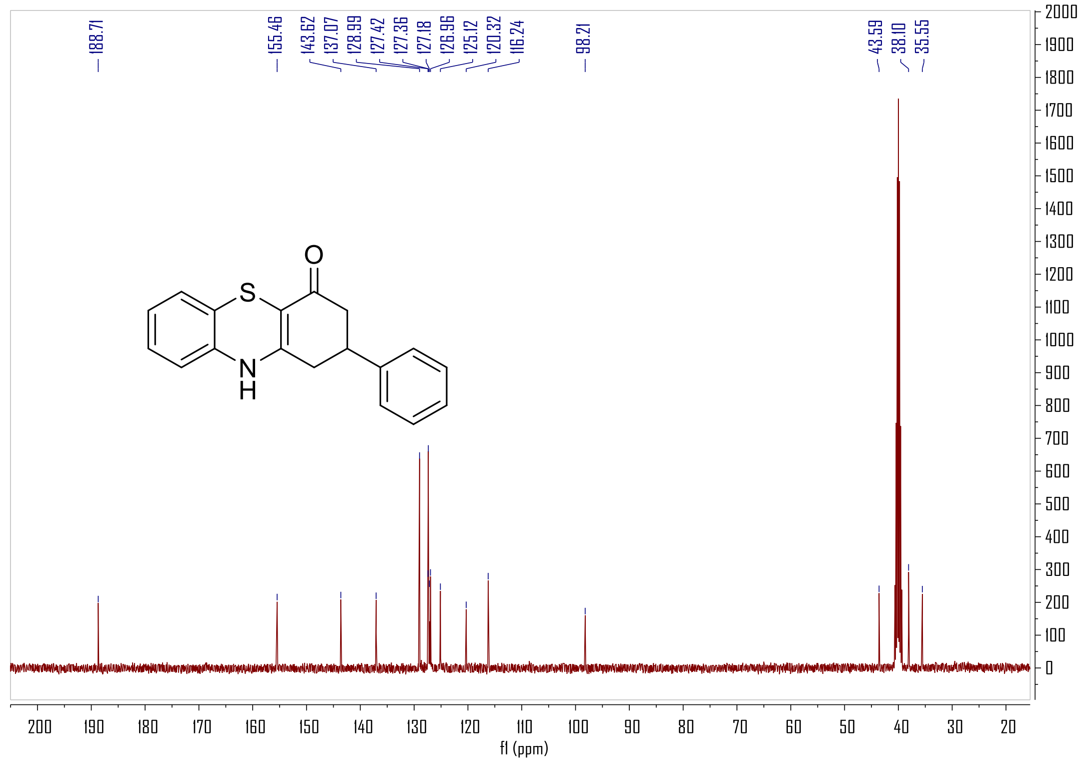

2,3-dihydro-1H-phenothiazin-4(10H)-one (3m)

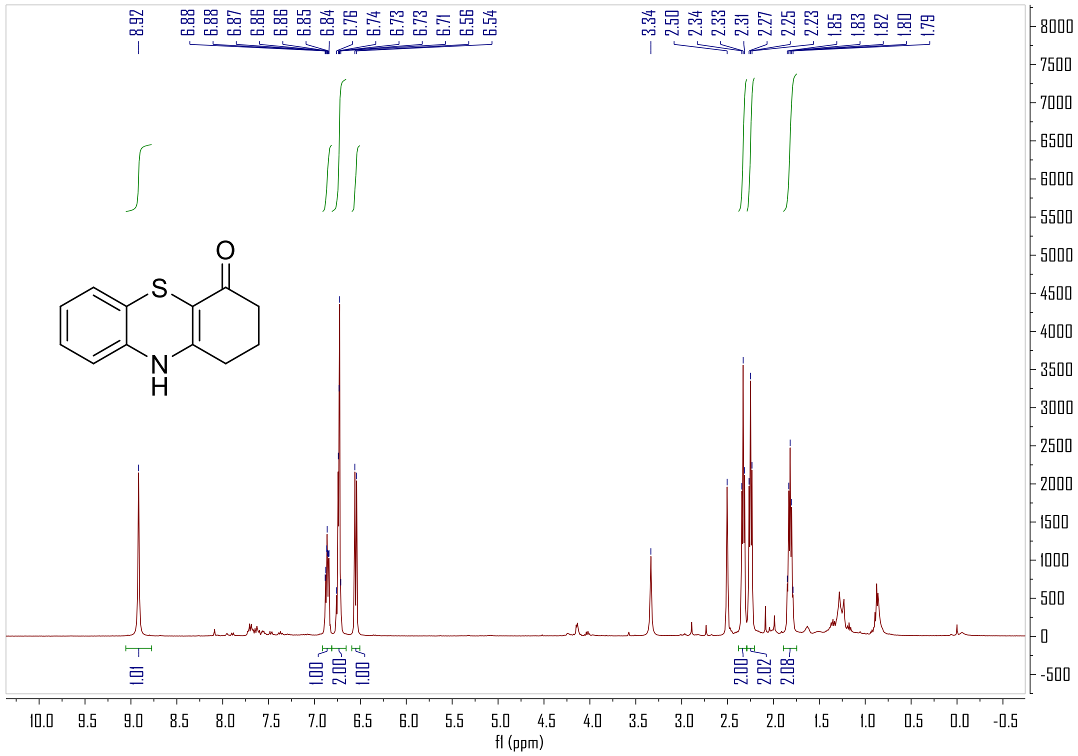

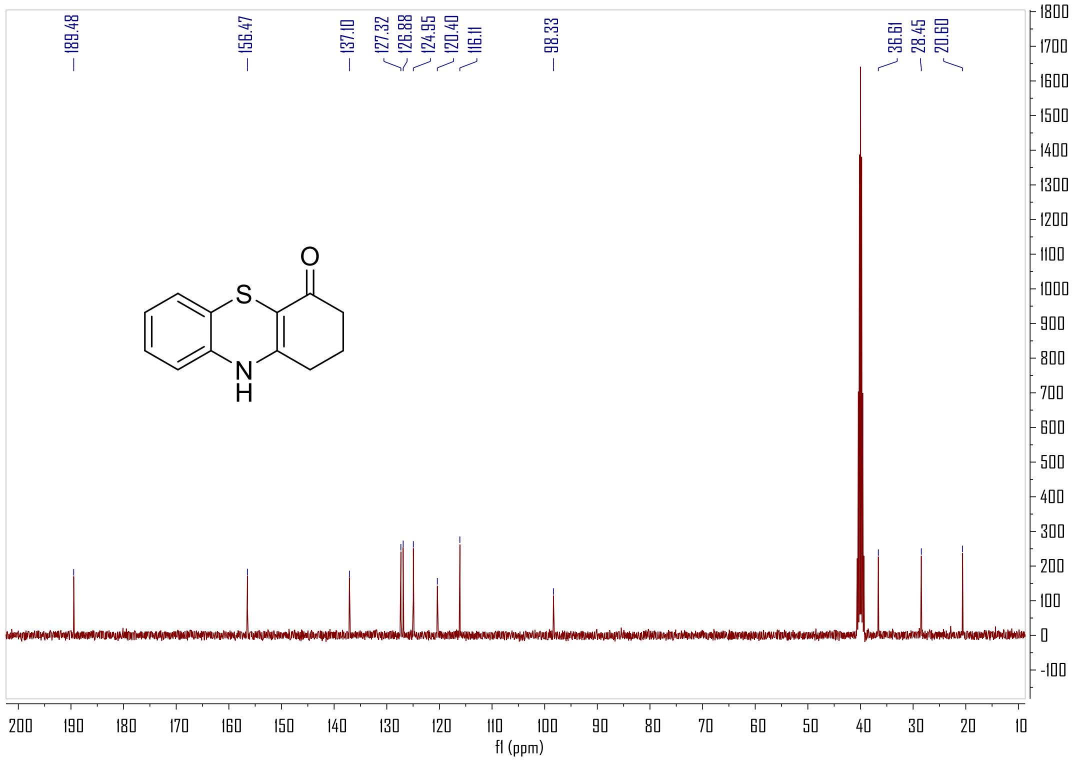

8-bromo-2,2-dimethyl-2,3-dihydro-1H-phenothiazin-4(10H)-one (3o)

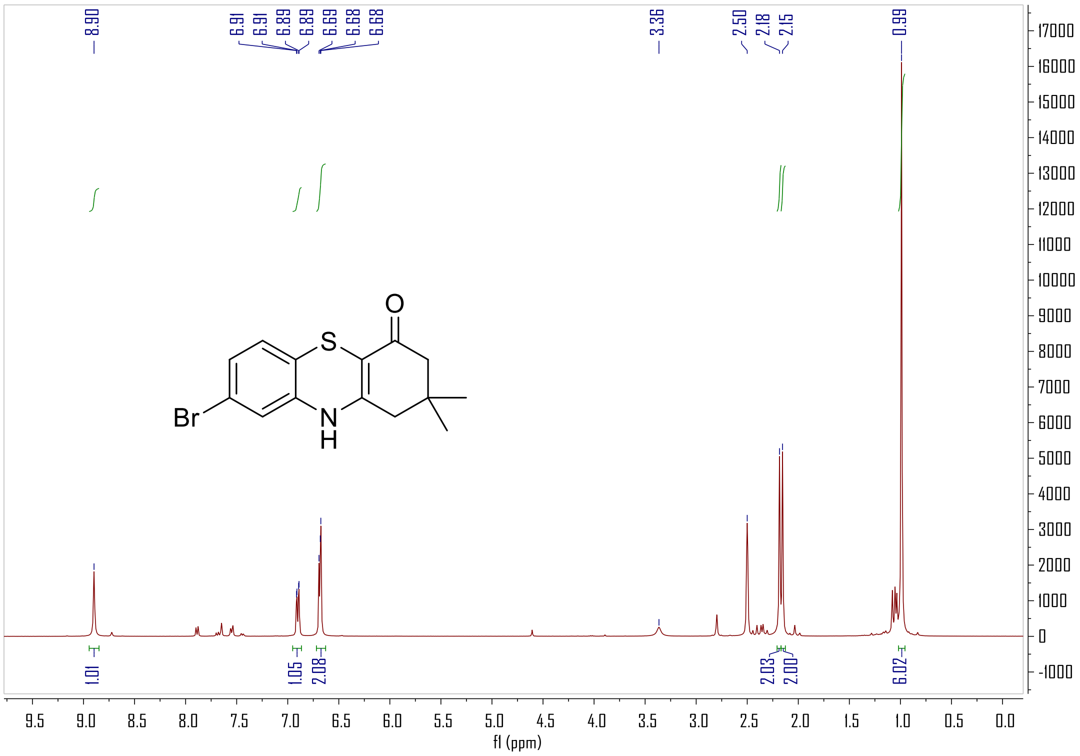

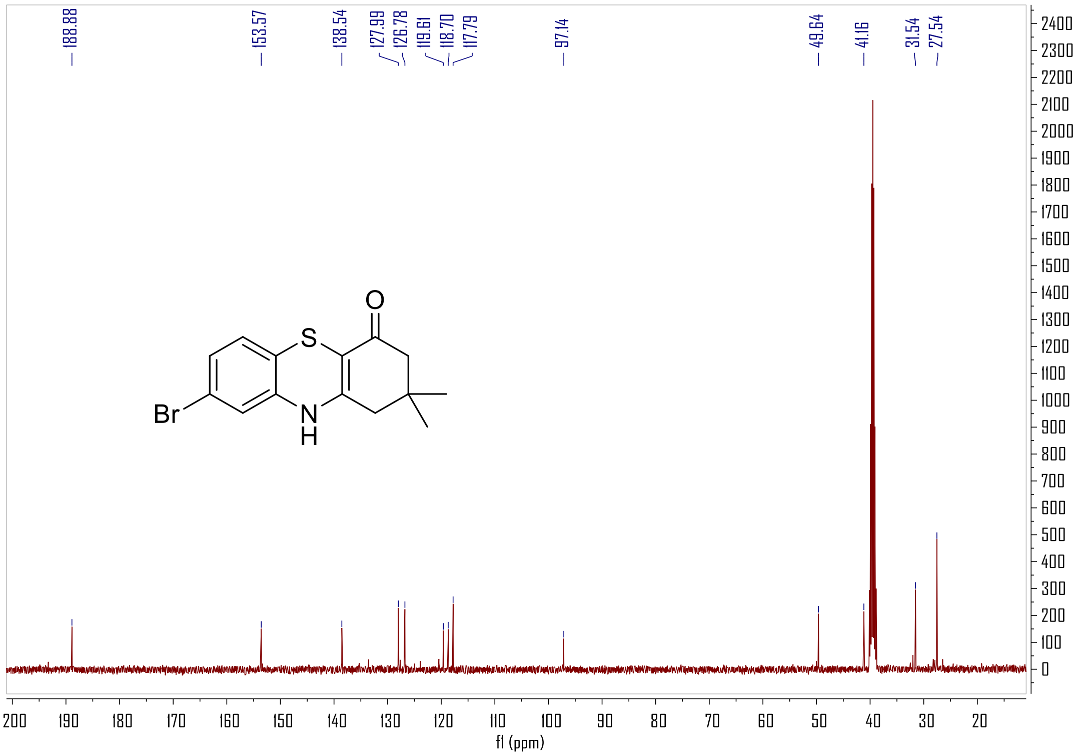

9-bromo-2,2-dimethyl-2,3-dihydro-1H-phenothiazin-4(10H)-one (3p)

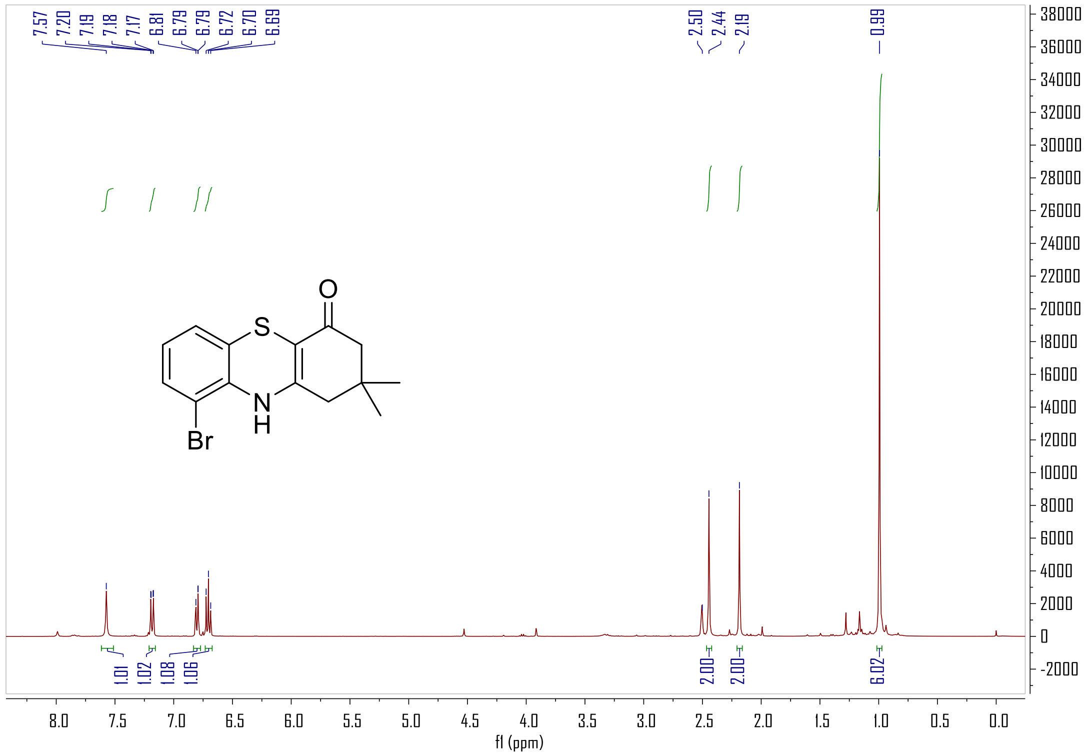

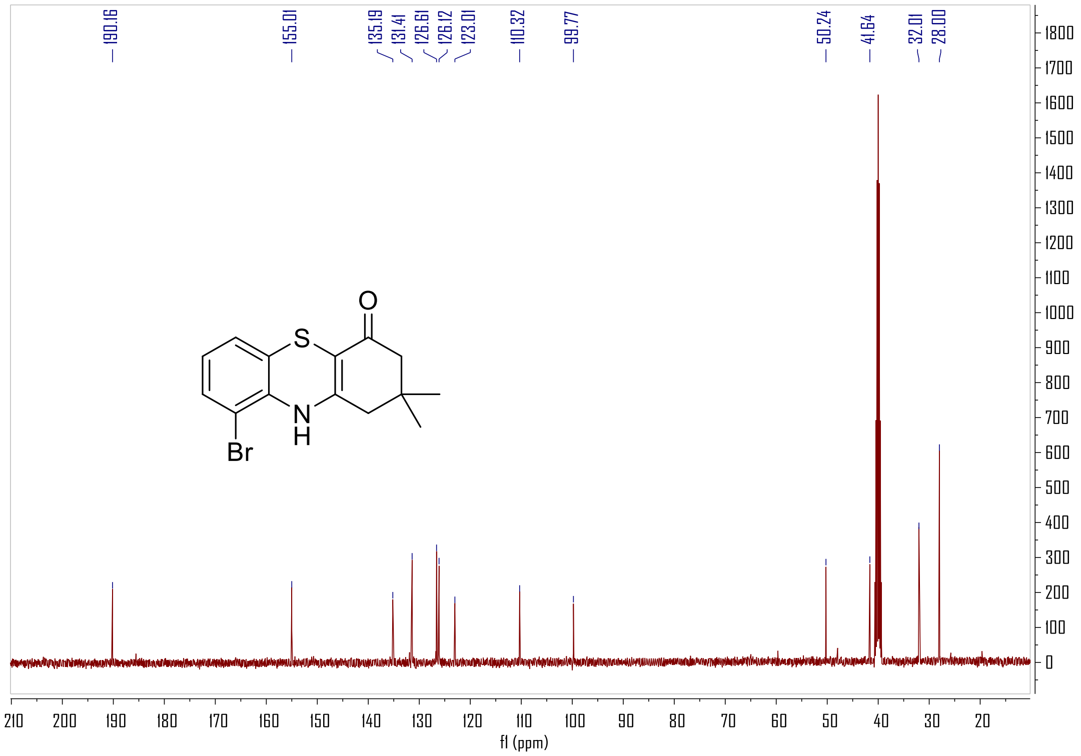

2-phenyl-4H-thiochromen-4-one (5a)

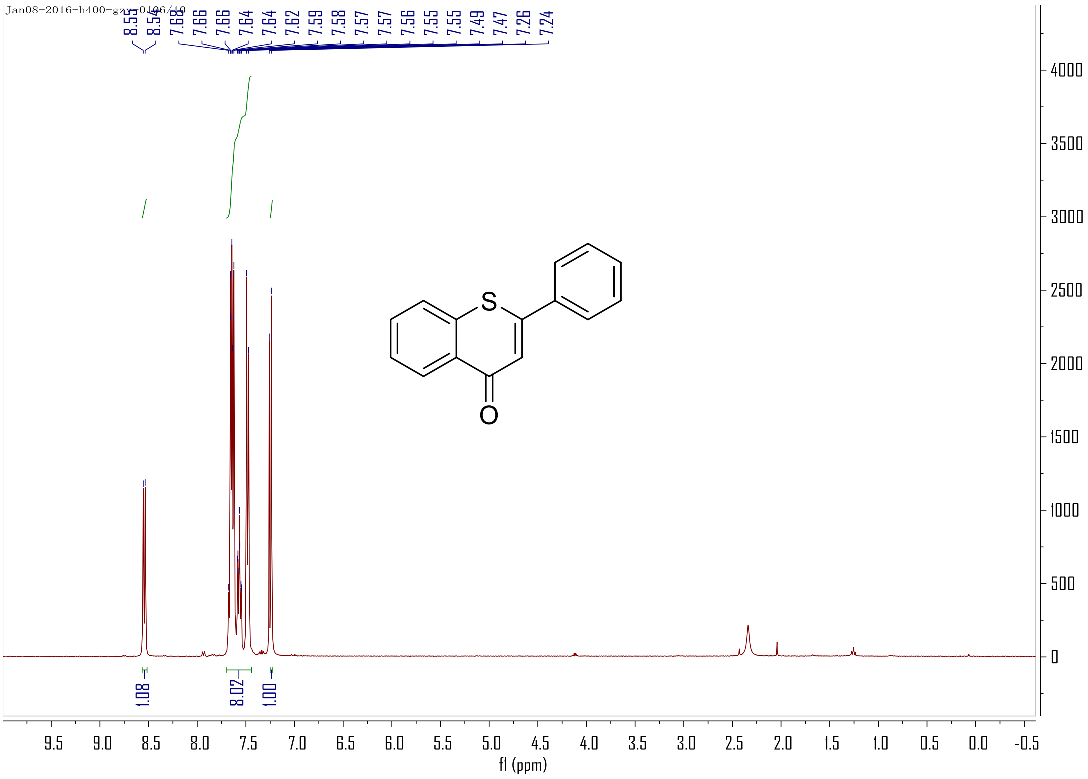

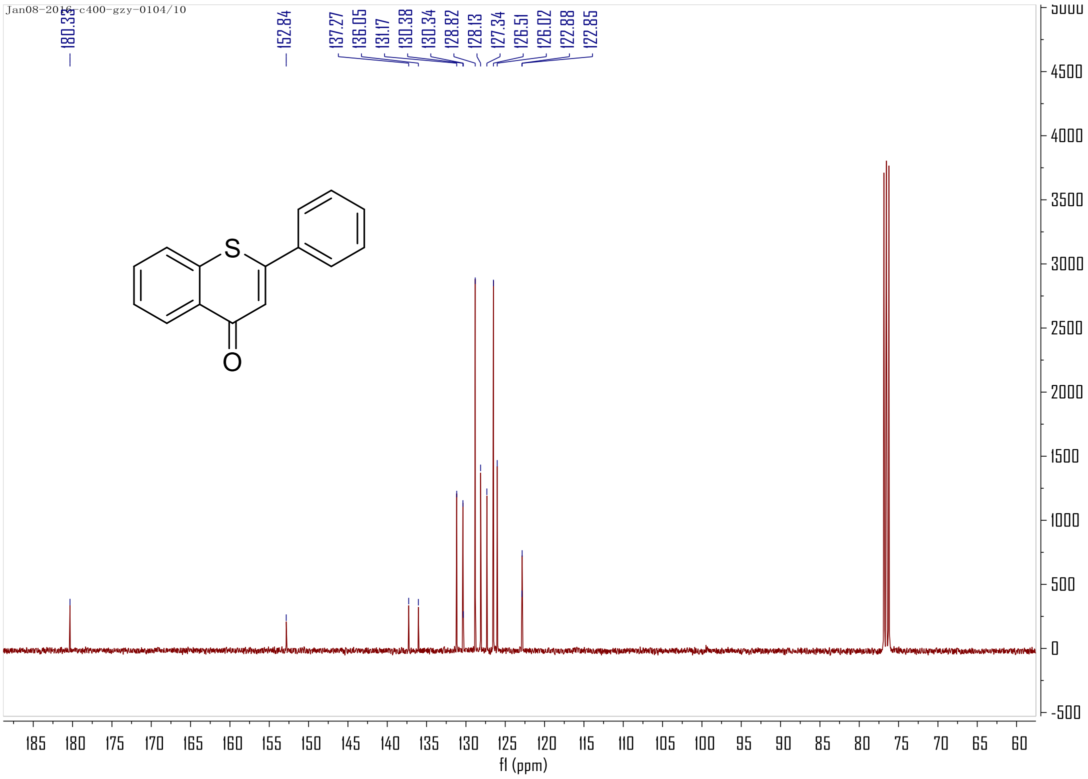

2-p-tolyl-4H-thiochromen-4-one (5b)

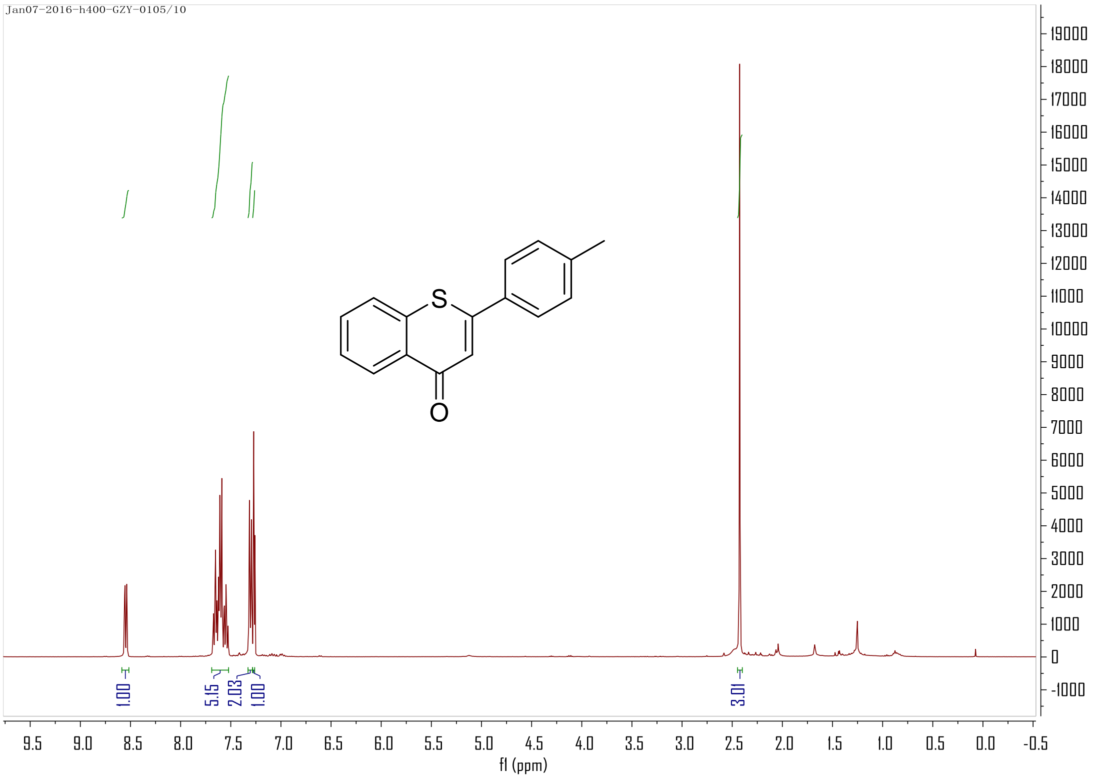

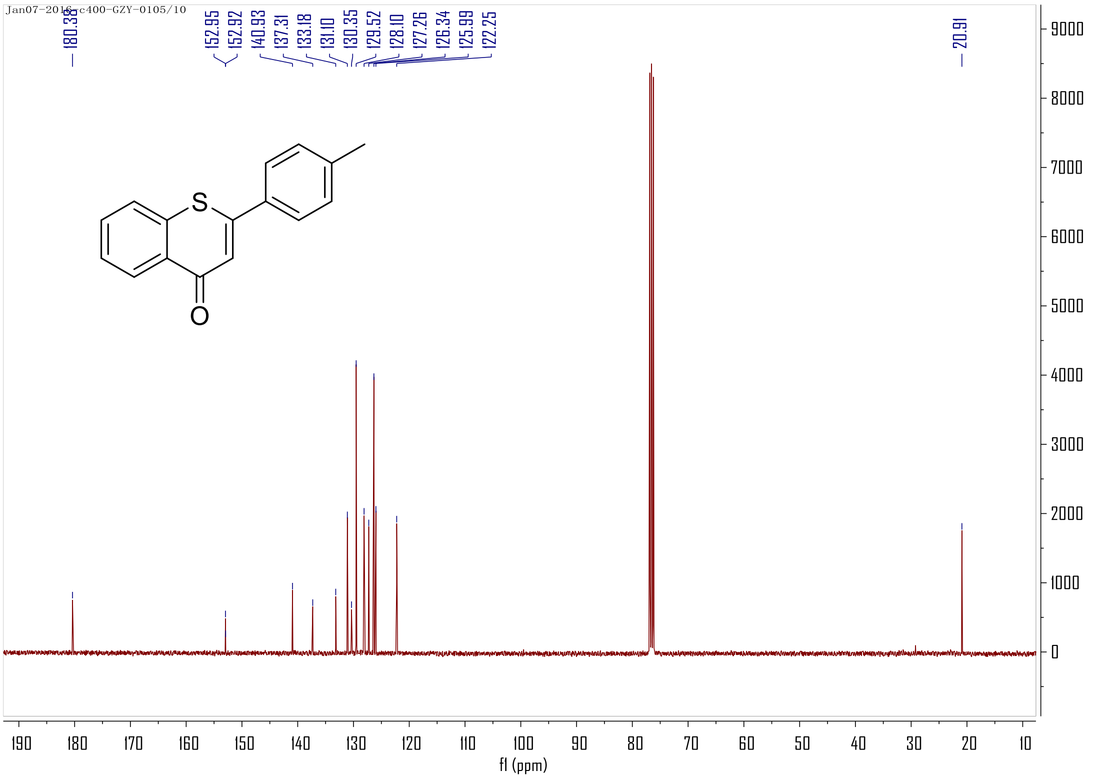

2-(4-fluorophenyl)-4H-thiochromen-4-one (5c)

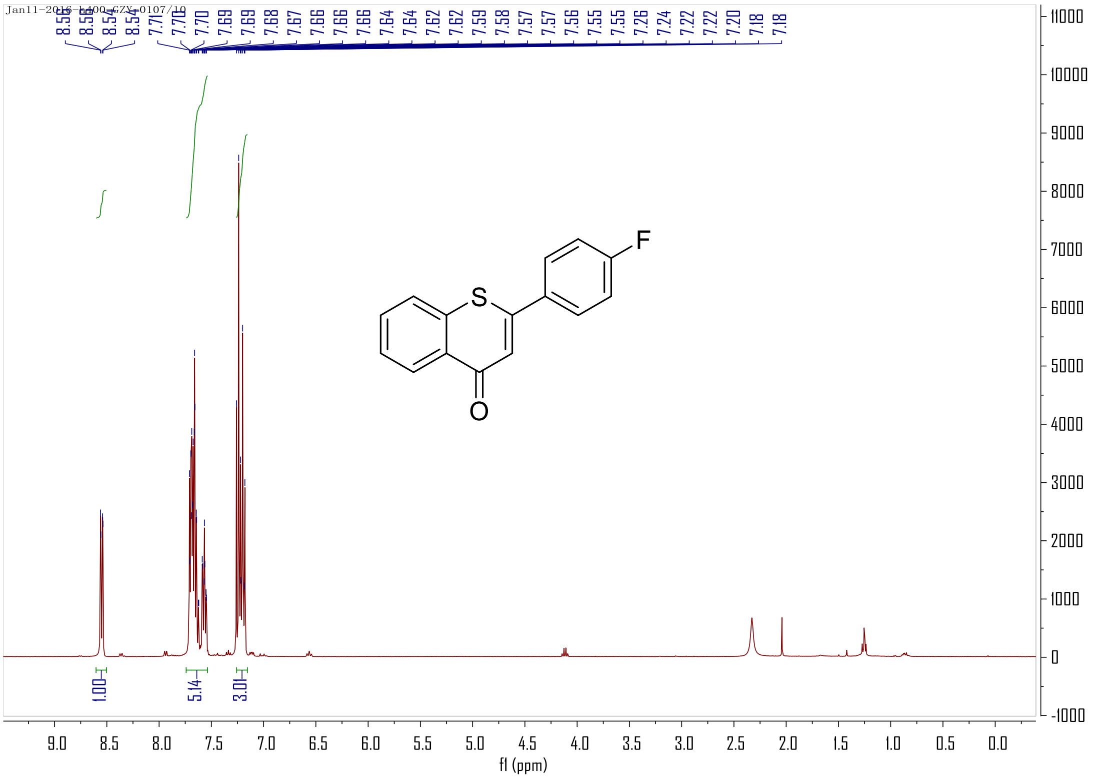

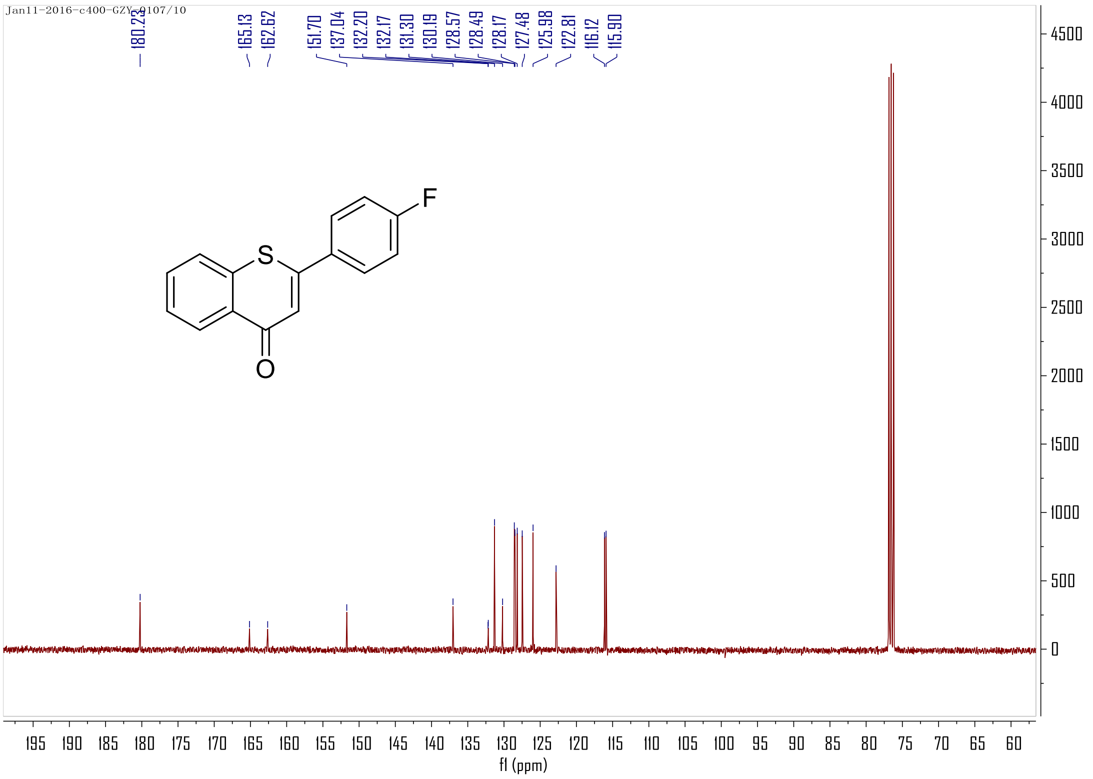

2-(4-chlorophenyl)-4H-thiochromen-4-one (5d)

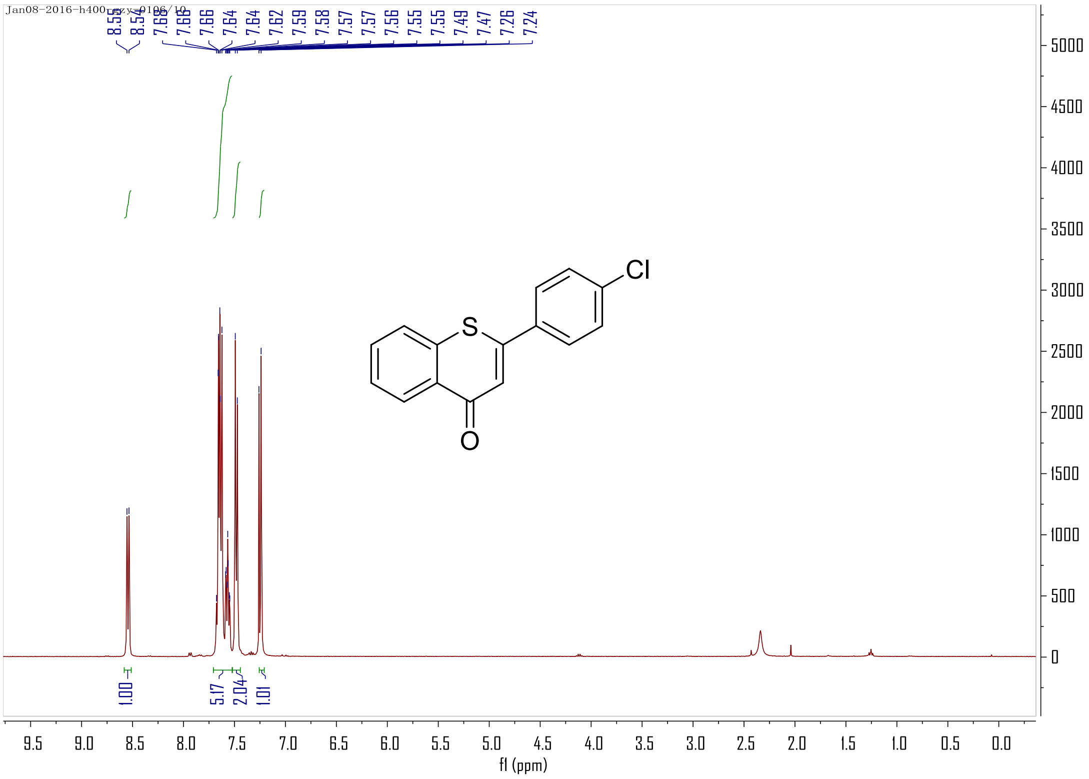

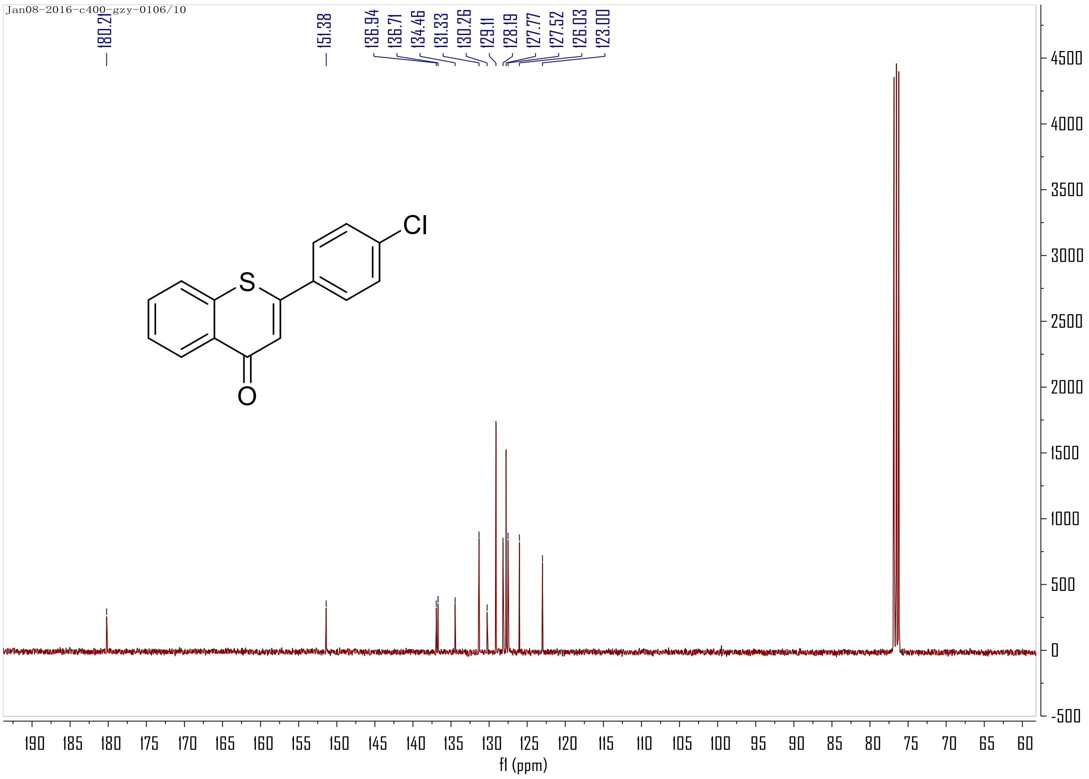

2-(thiophen-2-yl)-4H-thiochromen-4-one (5e)

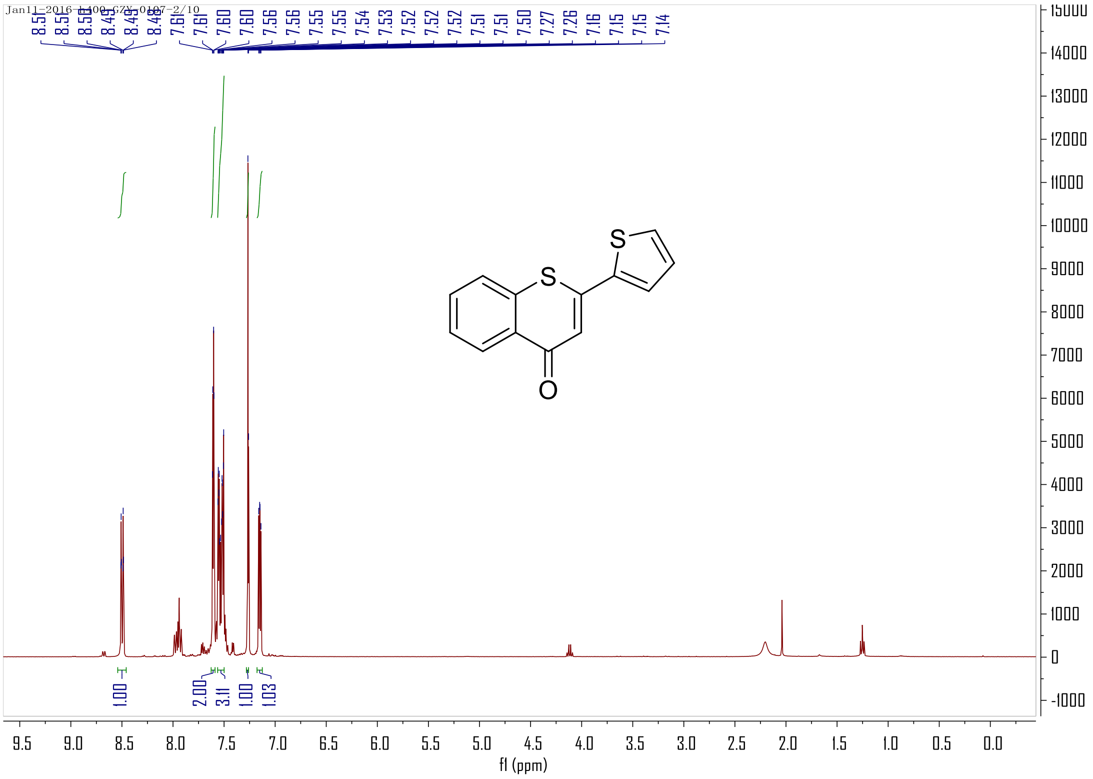

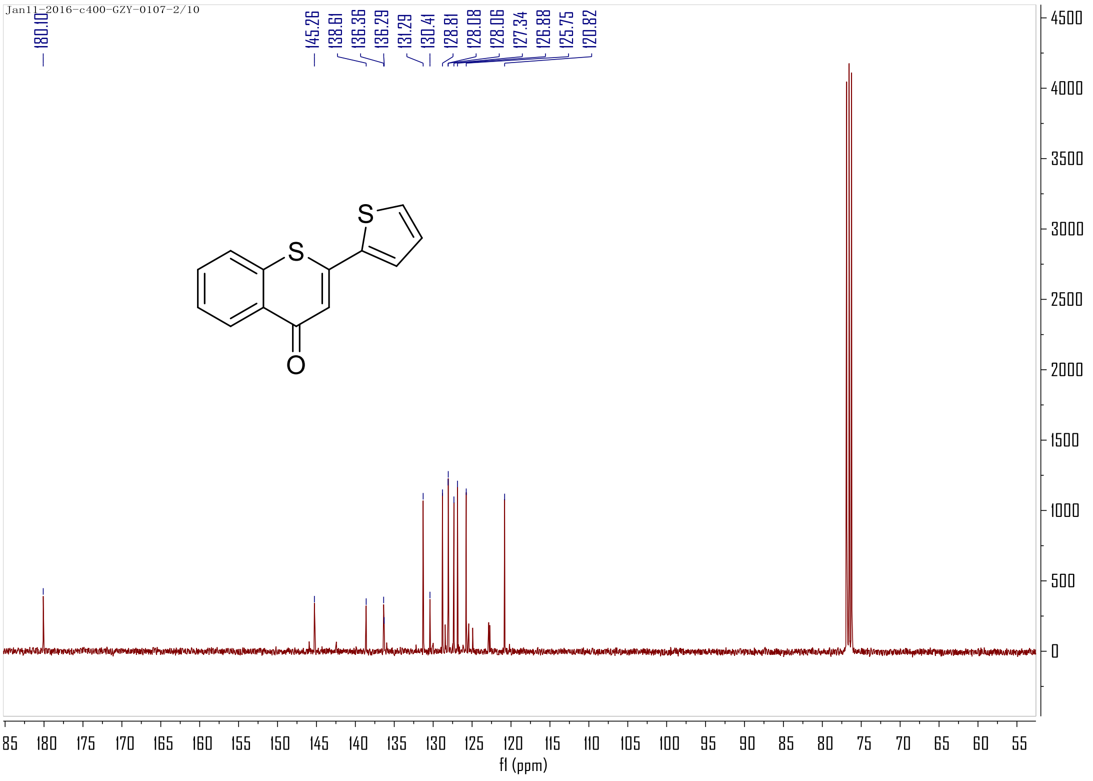

X-ray crystallographic data of 3a

```
data_b150114a_0ma_a_a_a
_audit_creation_date              2015-01-15
_audit_creation_method
;
  Olex2 1.2-ac2
  (compiled 2012.03.06 svn.r2239, GUI svn.r4109)
;
_publ_contact_author_address      ?
_publ_contact_author_email        ?
_publ_contact_author_name         ''
_publ_contact_author_phone        ?
_chemical_name_common             ?
_chemical_name_systematic
;
 ?
;
_chemical_formula_moiety          'C14 H15 N O S'
_chemical_formula_sum             'C14 H15 N O S'
_chemical_formula_weight          245.33
_chemical_melting_point           ?
loop_
  _atom_type_symbol
  _atom_type_description
  _atom_type_scat_dispersion_real
  _atom_type_scat_dispersion_imag
  _atom_type_scat_source
 'C' 'C' 0.0033 0.0016 'International Tables Vol C Tables 4.2.6.8 and 6.1.1.4'
 'H' 'H' 0.0000 0.0000 'International Tables Vol C Tables 4.2.6.8 and 6.1.1.4'
 'N' 'N' 0.0061 0.0033 'International Tables Vol C Tables 4.2.6.8 and 6.1.1.4'
 'O' 'O' 0.0106 0.0060 'International Tables Vol C Tables 4.2.6.8 and 6.1.1.4'
 'S' 'S' 0.1246 0.1234 'International Tables Vol C Tables 4.2.6.8 and 6.1.1.4'

_space_group_crystal_system       'monoclinic'
_space_group_IT_number            14
_space_group_name_H-M_alt         'P 1 21/c 1'
_space_group_name_Hall            '-P 2ybc'
loop_
  _space_group_symop_id
  _space_group_symop_operation_xyz
 1 'x, y, z'
 2 '-x, y+1/2, -z+1/2'
 3 '-x, -y, -z'
```

4 'x, -y-1/2, z-1/2'

_cell_length_a 17.5010(19)
_cell_length_b 5.6716(6)
_cell_length_c 12.6807(14)
_cell_angle_alpha 90.00
_cell_angle_beta 104.641(3)
_cell_angle_gamma 90.00
_cell_volume 1217.8(2)
_cell_formula_units_Z 4
_cell_measurement_reflns_used 8403
_cell_measurement_temperature 273(2)
_cell_measurement_theta_max 28.07
_cell_measurement_theta_min 3.23
_exptl_absorpt_coefficient_mu 0.248
_exptl_absorpt_correction_T_max 0.9877
_exptl_absorpt_correction_T_min 0.8861
_exptl_absorpt_correction_type 'none'
_exptl_absorpt_process_details sadabs
_exptl_crystal_colour 'orange'
_exptl_crystal_density_diffrn 1.338
_exptl_crystal_density_meas ?
_exptl_crystal_density_method 'not measured'
_exptl_crystal_description 'plate'
_exptl_crystal_F_000 520
_exptl_crystal_size_max 0.5
_exptl_crystal_size_mid 0.4
_exptl_crystal_size_min 0.05
_exptl_special_details sadabs
_diffrn_reflns_av_R_equivalents 0.0946
_diffrn_reflns_av_unetI/netI 0.0518
_diffrn_reflns_limit_h_max 21
_diffrn_reflns_limit_h_min -21
_diffrn_reflns_limit_k_max 7
_diffrn_reflns_limit_k_min -7
_diffrn_reflns_limit_l_max 15
_diffrn_reflns_limit_l_min -15
_diffrn_reflns_number 19390
_diffrn_reflns_theta_full 26.37
_diffrn_reflns_theta_max 26.37
_diffrn_reflns_theta_min 3.23
_diffrn_ambient_temperature 273.15
_diffrn_detector_area_resol_mean ?
_diffrn_measured_fraction_theta_full 0.999

_diffrn_measured_fraction_theta_max 0.999
_diffrn_measurement_device_type 'Bruker APEX-II CCD'
_diffrn_measurement_method '\f and \w scans'
_diffrn_radiation_monochromator graphite
_diffrn_radiation_type Mo
_diffrn_radiation_wavelength 0.71073
_diffrn_source 'fine-focus sealed tube'
_diffrn_source_current 1.0
_diffrn_source_power 0.05
_diffrn_source_voltage 50.0
_diffrn_standards_decay_% ?
_diffrn_standards_interval_count ?
_diffrn_standards_interval_time ?
_diffrn_standards_number 0
_reflns_number_gt 2001
_reflns_number_total 2486
_reflns_threshold_expression >2sigma(I)
_computing_cell_refinement ?
_computing_data_collection ?
_computing_data_reduction ?
_computing_molecular_graphics
;
O. V. Dolomanov, L. J. Bourhis, R. J. Gildea, J. A. K. Howard and H. Puschmann,
OLEX2: a complete structure solution, refinement and analysis program.
J. Appl. Cryst. (2009). 42, 339-341.
;
_computing_publication_material
;
O. V. Dolomanov, L. J. Bourhis, R. J. Gildea, J. A. K. Howard and H. Puschmann,
OLEX2: a complete structure solution, refinement and analysis program.
J. Appl. Cryst. (2009). 42, 339-341.
;
_computing_structure_refinement
;
XL, G.M. Sheldrick, Acta Cryst.
(2008). A64, 112-122
;
_computing_structure_solution
;
XS, G.M. Sheldrick, Acta Cryst.
(2008). A64, 112-122
;
_refine_diff_density_max 0.375
_refine_diff_density_min -0.300

_refine_diff_density_rms 0.060
_refine_ls_extinction_coef ?
_refine_ls_extinction_method none
_refine_ls_goodness_of_fit_ref 1.065
_refine_ls_hydrogen_treatment mixed
_refine_ls_matrix_type full
_refine_ls_number_parameters 156
_refine_ls_number_reflns 2486
_refine_ls_number_restraints 0
_refine_ls_R_factor_all 0.0618
_refine_ls_R_factor_gt 0.0467
_refine_ls_restrained_S_all 1.065
_refine_ls_shift/su_max 0.000
_refine_ls_shift/su_mean 0.000
_refine_ls_structure_factor_coef Fsqd
_refine_ls_weighting_details
'calc w=1/[\s^2^(Fo^2^)+(0.1000P)^2^+0.0000P] where P=(Fo^2^+2Fc^2^)/3'
_refine_ls_weighting_scheme calc
_refine_ls_wR_factor_gt 0.1396
_refine_ls_wR_factor_ref 0.1504
_refine_special_details
;
Refinement of F^2^ against ALL reflections. The weighted R-factor wR and goodness of fit S are based on F^2^, conventional R-factors R are based on F, with F set to zero for negative F^2^. The threshold expression of F^2^ > 2sigma(F^2^) is used only for calculating R-factors(gt) etc. and is not relevant to the choice of reflections for refinement. R-factors based on F^2^ are statistically about twice as large as those based on F, and R-factors based on ALL data will be even larger.
;
_atom_sites_solution_hydrogens geom
_atom_sites_solution_primary direct
_atom_sites_solution_secondary difmap
loop_
_atom_site_label
_atom_site_type_symbol
_atom_site_fract_x
_atom_site_fract_y
_atom_site_fract_z
_atom_site_U_iso_or_equiv
_atom_site_adp_type
_atom_site_occupancy
_atom_site_symmetry_multiplicity
_atom_site_calc_flag

_atom_site_refinement_flags
_atom_site_disorder_assembly
_atom_site_disorder_group
S1 S 0.66585(3) 0.67870(10) 0.56221(4) 0.0294(2) Uani 1 1 d . . .
O1 O 0.76587(9) 1.0537(2) 0.52787(11) 0.0311(4) Uani 1 1 d . . .
N1 N 0.70945(9) 0.6175(3) 0.81490(13) 0.0234(4) Uani 1 1 d . . .
H1 H 0.7219 0.6001 0.8845 0.028 Uiso 1 1 calc R . .
C1 C 0.62796(11) 0.4737(3) 0.64064(16) 0.0209(4) Uani 1 1 d . . .
C2 C 0.57177(12) 0.3136(4) 0.58626(18) 0.0268(5) Uani 1 1 d . . .
H2 H 0.5540 0.3215 0.5108 0.032 Uiso 1 1 calc R . .
C3 C 0.54170(12) 0.1427(4) 0.64221(19) 0.0306(5) Uani 1 1 d . . .
H3 H 0.5043 0.0362 0.6043 0.037 Uiso 1 1 calc R . .
C4 C 0.56704(12) 0.1301(4) 0.75404(19) 0.0292(5) Uani 1 1 d . . .
H4 H 0.5471 0.0148 0.7919 0.035 Uiso 1 1 calc R . .
C5 C 0.62240(11) 0.2901(4) 0.80981(17) 0.0247(5) Uani 1 1 d . . .
H5 H 0.6394 0.2822 0.8854 0.030 Uiso 1 1 calc R . .
C6 C 0.65282(11) 0.4620(3) 0.75421(16) 0.0197(4) Uani 1 1 d . . .
C7 C 0.74608(11) 0.7909(3) 0.77481(16) 0.0194(4) Uani 1 1 d . . .
C8 C 0.73298(11) 0.8379(3) 0.66616(16) 0.0203(4) Uani 1 1 d . . .
C9 C 0.77528(11) 1.0186(3) 0.62684(16) 0.0208(4) Uani 1 1 d . . .
C10 C 0.83154(12) 1.1714(3) 0.70850(17) 0.0242(5) Uani 1 1 d . . .
H10A H 0.8031 1.3081 0.7245 0.029 Uiso 1 1 calc R . .
H10B H 0.8728 1.2272 0.6760 0.029 Uiso 1 1 calc R . .
C11 C 0.86983(11) 1.0451(3) 0.81535(16) 0.0210(4) Uani 1 1 d . . .
C12 C 0.80381(11) 0.9326(3) 0.85829(16) 0.0224(5) Uani 1 1 d . . .
H12A H 0.8275 0.8310 0.9194 0.027 Uiso 1 1 calc R . .
H12B H 0.7754 1.0563 0.8851 0.027 Uiso 1 1 calc R . .
C13 C 0.92689(12) 0.8575(4) 0.79545(19) 0.0311(5) Uani 1 1 d . . .
H13A H 0.8993 0.7501 0.7403 0.047 Uiso 1 1 calc R . .
H13B H 0.9484 0.7726 0.8618 0.047 Uiso 1 1 calc R . .
H13C H 0.9690 0.9315 0.7717 0.047 Uiso 1 1 calc R . .
C14 C 0.91432(13) 1.2202(4) 0.90020(18) 0.0301(5) Uani 1 1 d . . .
H14A H 0.9549 1.2954 0.8737 0.045 Uiso 1 1 calc R . .
H14B H 0.9378 1.1381 0.9667 0.045 Uiso 1 1 calc R . .
H14C H 0.8782 1.3371 0.9136 0.045 Uiso 1 1 calc R . .

loop_
_atom_site_aniso_label
_atom_site_aniso_U_11
_atom_site_aniso_U_22
_atom_site_aniso_U_33
_atom_site_aniso_U_23
_atom_site_aniso_U_13
_atom_site_aniso_U_12

S1 0.0385(4) 0.0347(4) 0.0145(3) -0.0012(2) 0.0055(2) -0.0126(2)
O1 0.0437(9) 0.0336(8) 0.0175(8) 0.0018(6) 0.0105(7) -0.0069(7)
N1 0.0270(9) 0.0304(9) 0.0128(9) -0.0001(7) 0.0048(7) -0.0059(7)
C1 0.0190(10) 0.0242(10) 0.0210(11) -0.0008(8) 0.0080(8) 0.0025(8)
C2 0.0249(11) 0.0344(12) 0.0221(11) -0.0053(9) 0.0080(9) -0.0014(9)
C3 0.0280(11) 0.0324(12) 0.0338(13) -0.0110(10) 0.0123(10) -0.0073(9)
C4 0.0293(11) 0.0278(11) 0.0356(13) 0.0018(10) 0.0173(10) -0.0044(9)
C5 0.0239(10) 0.0309(11) 0.0219(11) 0.0004(9) 0.0102(9) 0.0003(8)
C6 0.0192(10) 0.0223(10) 0.0195(10) -0.0026(8) 0.0086(8) 0.0004(8)
C7 0.0208(10) 0.0212(10) 0.0182(10) -0.0001(8) 0.0085(8) 0.0030(8)
C8 0.0200(10) 0.0229(10) 0.0178(11) -0.0018(8) 0.0043(8) 0.0001(8)
C9 0.0230(10) 0.0219(10) 0.0183(11) 0.0004(8) 0.0070(8) 0.0030(8)
C10 0.0270(11) 0.0215(10) 0.0247(12) -0.0003(8) 0.0078(9) -0.0021(8)
C11 0.0221(10) 0.0217(10) 0.0191(10) -0.0012(8) 0.0051(8) -0.0002(8)
C12 0.0235(10) 0.0256(10) 0.0186(10) 0.0000(8) 0.0062(8) 0.0002(8)
C13 0.0246(11) 0.0315(12) 0.0375(14) -0.0016(10) 0.0084(10) 0.0032(9)
C14 0.0289(11) 0.0297(11) 0.0301(13) -0.0031(9) 0.0048(9) -0.0062(9)

_geom_special_details
;
All esds (except the esd in the dihedral angle between two l.s. planes)
are estimated using the full covariance matrix. The cell esds are taken
into account individually in the estimation of esds in distances, angles
and torsion angles; correlations between esds in cell parameters are only
used when they are defined by crystal symmetry. An approximate (isotropic)
treatment of cell esds is used for estimating esds involving l.s. planes.
;
loop_
_geom_bond_atom_site_label_1
_geom_bond_atom_site_label_2
_geom_bond_distance
_geom_bond_site_symmetry_2
_geom_bond_publ_flag
S1 C1 1.7648(19) . ?
S1 C8 1.775(2) . ?
O1 C9 1.240(2) . ?
N1 H1 0.8600 . ?
N1 C6 1.402(2) . ?
N1 C7 1.342(2) . ?
C1 C2 1.387(3) . ?
C1 C6 1.397(3) . ?
C2 H2 0.9300 . ?
C2 C3 1.381(3) . ?
C3 H3 0.9300 . ?

C3 C4 1.377(3) . ?
C4 H4 0.9300 . ?
C4 C5 1.384(3) . ?
C5 H5 0.9300 . ?
C5 C6 1.386(3) . ?
C7 C8 1.365(3) . ?
C7 C12 1.499(3) . ?
C8 C9 1.426(3) . ?
C9 C10 1.509(3) . ?
C10 H10A 0.9700 . ?
C10 H10B 0.9700 . ?
C10 C11 1.529(3) . ?
C11 C12 1.535(3) . ?
C11 C13 1.523(3) . ?
C11 C14 1.525(3) . ?
C12 H12A 0.9700 . ?
C12 H12B 0.9700 . ?
C13 H13A 0.9600 . ?
C13 H13B 0.9600 . ?
C13 H13C 0.9600 . ?
C14 H14A 0.9600 . ?
C14 H14B 0.9600 . ?
C14 H14C 0.9600 . ?

loop_
_geom_angle_atom_site_label_1
_geom_angle_atom_site_label_2
_geom_angle_atom_site_label_3
_geom_angle
_geom_angle_site_symmetry_1
_geom_angle_site_symmetry_3
_geom_angle_publ_flag
C1 S1 C8 100.91(9) . . ?
C6 N1 H1 116.9 . . ?
C7 N1 H1 116.9 . . ?
C7 N1 C6 126.29(17) . . ?
C2 C1 S1 118.11(15) . . ?
C2 C1 C6 118.59(18) . . ?
C6 C1 S1 123.29(15) . . ?
C1 C2 H2 119.4 . . ?
C3 C2 C1 121.2(2) . . ?
C3 C2 H2 119.4 . . ?
C2 C3 H3 120.0 . . ?
C4 C3 C2 120.0(2) . . ?

C4 C3 H3 120.0 . . ?
C3 C4 H4 120.2 . . ?
C3 C4 C5 119.60(19) . . ?
C5 C4 H4 120.2 . . ?
C4 C5 H5 119.7 . . ?
C4 C5 C6 120.7(2) . . ?
C6 C5 H5 119.7 . . ?
C1 C6 N1 121.92(17) . . ?
C5 C6 N1 118.17(18) . . ?
C5 C6 C1 119.90(18) . . ?
N1 C7 C8 123.44(18) . . ?
N1 C7 C12 115.25(17) . . ?
C8 C7 C12 121.31(17) . . ?
C7 C8 S1 124.13(15) . . ?
C7 C8 C9 121.59(17) . . ?
C9 C8 S1 114.25(15) . . ?
O1 C9 C8 121.44(18) . . ?
O1 C9 C10 119.93(17) . . ?
C8 C9 C10 118.62(17) . . ?
C9 C10 H10A 108.8 . . ?
C9 C10 H10B 108.8 . . ?
C9 C10 C11 113.75(16) . . ?
H10A C10 H10B 107.7 . . ?
C11 C10 H10A 108.8 . . ?
C11 C10 H10B 108.8 . . ?
C10 C11 C12 107.97(16) . . ?
C13 C11 C10 109.82(17) . . ?
C13 C11 C12 110.60(16) . . ?
C13 C11 C14 109.41(16) . . ?
C14 C11 C10 110.58(15) . . ?
C14 C11 C12 108.44(16) . . ?
C7 C12 C11 113.94(16) . . ?
C7 C12 H12A 108.8 . . ?
C7 C12 H12B 108.8 . . ?
C11 C12 H12A 108.8 . . ?
C11 C12 H12B 108.8 . . ?
H12A C12 H12B 107.7 . . ?
C11 C13 H13A 109.5 . . ?
C11 C13 H13B 109.5 . . ?
C11 C13 H13C 109.5 . . ?
H13A C13 H13B 109.5 . . ?
H13A C13 H13C 109.5 . . ?
H13B C13 H13C 109.5 . . ?
C11 C14 H14A 109.5 . . ?

C11 C14 H14B 109.5 . . ?
C11 C14 H14C 109.5 . . ?
H14A C14 H14B 109.5 . . ?
H14A C14 H14C 109.5 . . ?
H14B C14 H14C 109.5 . . ?

loop_
_geom_hbond_atom_site_label_D
_geom_hbond_atom_site_label_H
_geom_hbond_atom_site_label_A
_geom_hbond_distance_DH
_geom_hbond_distance_HA
_geom_hbond_distance_DA
_geom_hbond_angle_DHA
_geom_hbond_site_symmetry_A
N1 H1 O1 0.86 1.99 2.804(2) 158.2 4_576
